# NetGen: a novel network-based probabilistic generative model for gene set functional enrichment analysis

**DOI:** 10.1186/s12918-017-0456-7

**Published:** 2017-09-21

**Authors:** Duanchen Sun, Yinliang Liu, Xiang-Sun Zhang, Ling-Yun Wu

**Affiliations:** 1 Institute of Applied Mathematics, Academy of Mathematics and Systems Science, Chinese Academy of Sciences, Beijing, 100190 China; 2 National Center for Mathematics and Interdisciplinary Sciences, Chinese Academy of Sciences, Beijing, 100190 China; 3 School of Mathematical Sciences, University of Chinese Academy of Sciences, Beijing, 100049 China

**Keywords:** Gene ontology, Enrichment analysis, Network-based probabilistic generative model, Integer programming, Complex diseases

## Abstract

**Background:**

High-throughput experimental techniques have been dramatically improved and widely applied in the past decades. However, biological interpretation of the high-throughput experimental results, such as differential expression gene sets derived from microarray or RNA-seq experiments, is still a challenging task. Gene Ontology (GO) is commonly used in the functional enrichment studies. The GO terms identified via current functional enrichment analysis tools often contain direct parent or descendant terms in the GO hierarchical structure. Highly redundant terms make users difficult to analyze the underlying biological processes.

**Results:**

In this paper, a novel network-based probabilistic generative model, NetGen, was proposed to perform the functional enrichment analysis. An additional protein-protein interaction (PPI) network was explicitly used to assist the identification of significantly enriched GO terms. NetGen achieved a superior performance than the existing methods in the simulation studies. The effectiveness of NetGen was explored further on four real datasets. Notably, several GO terms which were not directly linked with the active gene list for each disease were identified. These terms were closely related to the corresponding diseases when accessed to the curated literatures. NetGen has been implemented in the R package CopTea publicly available at GitHub (http://github.com/wulingyun/CopTea/).

**Conclusion:**

Our procedure leads to a more reasonable and interpretable result of the functional enrichment analysis. As a novel term combination-based functional enrichment analysis method, NetGen is complementary to current individual term-based methods, and can help to explore the underlying pathogenesis of complex diseases.

**Electronic supplementary material:**

The online version of this article (doi:10.1186/s12918-017-0456-7) contains supplementary material, which is available to authorized users.

## Background

High-throughput experimental techniques, such as microarray, mass spectrometry and next-generation sequencing, have become indispensable tools for biological and medical researches. These high-throughput experiments usually generate large interesting gene lists as their final outputs, which share some certain characteristics. A large fraction of the gene outputs specifies the key biological functions underlying the studied samples. Therefore, interpreting the biological meaning of the similar characteristics and exploring the functional relationships among the selected genes are one of the important and challenging tasks in bioinformatics.

Gene Ontology (GO) project is a major bioinformatics initiative to produce a structured, dynamic, controlled vocabulary to describe key domains of molecular and cellular biology [1] and unify the representation of gene and gene product attributes [2, 3]. Due to the hierarchical structure of the GO, terms located at the top region often have more general molecular and cellular interpretation, and cover larger genes. On the other hand, terms at the bottom of the hierarchical structure represent more specific biological explanations. For example, ‘induction of apoptosis’ is a part of ‘apoptosis’, and the former is the most specific term. Therefore, genes annotated by the specific term are implicitly annotated by both its parent terms. As consequence, we cannot clearly know that which child term is a major reason to make the gene set significant, if the term ‘apoptosis’ is determined to be significantly enriched. So if the significant terms are chosen based on enrichment *p*-values, such as commonly used statistical methods, they may obscure other more important terms and make it hard to determine the most relevant explanations.

For addressing this issue, a variety of methods, aiming at finding the most involved functional relationships among the selected genes, have been developed during the past decades to perform the GO enrichment analysis. Basing on the model input (type of gene list) and the output (evaluation pattern of the identified GO terms), these methods can be briefly categorized into three classes (See Additional file 1, Table S1, Class I-III). The first class, represented by GOMiner [4], EASE [5], GOstat [6] onto-express [7], EnrichNet [8], MAPPFinder [9], etc., used a gene set as model input and output the significant level of each GO term, which was mainly based on the Fisher’s exact test [10]. A preset threshold value was usually selected by user to generate the gene list of interest. The second class, represented by GSEA [11], GLOBALTEST [12], SIGPATHWAY [13], PAGE [14], SAFE [15], EasyGO [16], etc., used a whole gene list with corresponding scores to evaluate each GO term. These methods combined the selection of differential expression genes and the enrichment analysis, and no need to preset a threshold for generating the gene list of interest. The major disadvantage of class II methods is its lacking of flexibility, for the scores are not easily accessible under sophisticated biological experiments. Class I and II both individually evaluated the terms, which brought a higher similarity and redundancy of identified terms. To make up this drawback, the third class, represented by DAVID [17], MGSA [18], GenGO [19], MCOA [20], eliminated the redundancy of enrichment analysis from the perspective of term set. Given a gene list as model input, one or more most enriched term sets were returned as model outputs. The terms in a term set may be similar or complementary. For example, DAVID [17] reduces the redundancy in the result of enrichment analysis by grouping similar terms into functional clusters.

In this paper, a novel network-based probabilistic generative model, NetGen, was proposed to perform the enrichment analysis. We followed the framework introduced in GenGO [19] that the gene list of interest was generated by some GO terms, which can retell the true story beneath the biological experiment. Lu, et al., mainly assumed that the active information of terms could passed to the annotated genes. Therefore, they defined a probabilistic model on the activation graph which contains both gene and GO nodes. By maximizing the likelihood of their model conditioned on the set of active genes, their final results shown that GenGO is prone to directly identify the combination of complementary (i.e. non-redundant) terms, and GenGO has a good performance when compared with other methods on both yeast and human GO database. Particularly, we provided a brand new perspective to consider this framework. In our model, an additional protein-protein interaction (PPI) network was explicitly used to assist the functional analysis. We assumed that the effect of active terms not only passed to the directly annotated genes, but also can affected the neighboring genes of the annotated genes in the PPI network. This supporting influence get weaker when the distances from the annotated genes get larger. Our procedure can lead to a more reasonable and explainable result of the functional analysis. Maximizing the log-likelihood estimation function can be formulated as a 0–1 integer programming problem. We used a greedy algorithm to identify the most enriched term combination.

During the past years, many methods that integrate the information of biological network have been developed to improve the performance of the enrichment analysis. For example, Wang, et al. [21] proposed a network ontology analysis (NOA) method to perform the GO enrichment analysis on biological networks. Network enrichment analysis (NEA) [22] extends the traditional overlap statistic in gene-set analysis to network links between genes in experimental output list and those in function terms. EnrichNet [8] first scores the distances between the gene list and reference gene set in the network using random walk with restart algorithm, then compares these scores with a background model to derive their final results. There are two reasons why we added the network information to assist the functional analysis. First, the GO annotation database is far from complete. Due to the underlying incompletion of GO annotation, some annotation links between GO terms and genes have not been established. Therefore, traditional functional analysis cannot identify these candidate terms. On the other hand, the interaction network was built based on the physical contacts of proteins. Proteins are prone to share a similar biological function, if their distance is short in the PPI network, which can be used to compensate for the incompletion of the GO annotation. Second, with the additional network information, our network-based generative model can simulate the upstream-downstream regulatory mechanism. Specifically, the neighboring genes and the directly annotated genes can be viewed as the downstream targets and the upstream regulators, respectively. In many cases, upstream regulators have only subtle expression variation therefore they may not be directly identified and emerge in the gene list of interest. However, through the directly annotated genes and network, the effect of active terms passes to the downstream genes, which may be observed and selected into the gene list of interest. The potential terms can be identified with a more reasonable and explainable result.

In this work, we first compared the performance of NetGen and classic individual term-based or term combination-based enrichment analysis methods in the simulation studies. NetGen achieved a superior performance than GenGO [19] and Fisher’s exact test [10], when the active gene list was generated under our assumption. We further explored the effectiveness of NetGen on four real datasets. Notably, we identified several terms which were not directly linked with the active gene list (Fisher’s exact test, *p* = 1) for each disease. These terms were closely related to the corresponding diseases according to the literature. All these pieces of evidence showed that NetGen is an efficient computational tool for functional enrichment analysis and can help to explore the underlying pathogenesis of complex diseases. NetGen has been compiled in the R package CopTea, which is available at GitHub (http://github.com/wulingyun/CopTea/) for users.

## Methods

### Network-based probabilistic generative model

In our network-based probabilistic generative model, the model input is the gene list of interest *G* (active gene list). We would like to identify the most enriched GO term set, which provides a reasonable biological explanation to *G*. Here our model assumed that *G* is generated by several unknown active GO terms, by which we can investigate and gain the insights into the related biological experiments or problems. Under this assumption, we completely modeled the generative process that propagate the active information from terms to genes and further through the biological network. In detail, our generative model can be explicated as follows.

First, some related GO terms are activated under the specific biological condition. The genes which are annotated by these active GO terms are defined as the core genes. Each core gene is activated (i.e. observed in biological experiments result *G*) with a relatively large probability *p*_1_. Second, we explicitly take the information propagation in biological network into consideration. Two genes with a relationship in biological network prone to have the similar functions. Generally speaking, the larger distance between two genes, the lower probability that they share the same biological functions (i.e. they are annotated by the same term). Therefore, we assumed that the genes which are close to the core genes in biological network do have a small chance to be activated, and the influences of core genes get weaker with the distances increase. In this paper, we only consider the direct neighbors of the core genes in biological network, which are defined as the peripheral genes. Each peripheral gene is activated with a relatively low probability *p*_2_. Last, due to the inevitable noises and errors in biological experiments, other genes also have a very low probability *q* to be picked up into the active gene list.

**Fig. 1:**
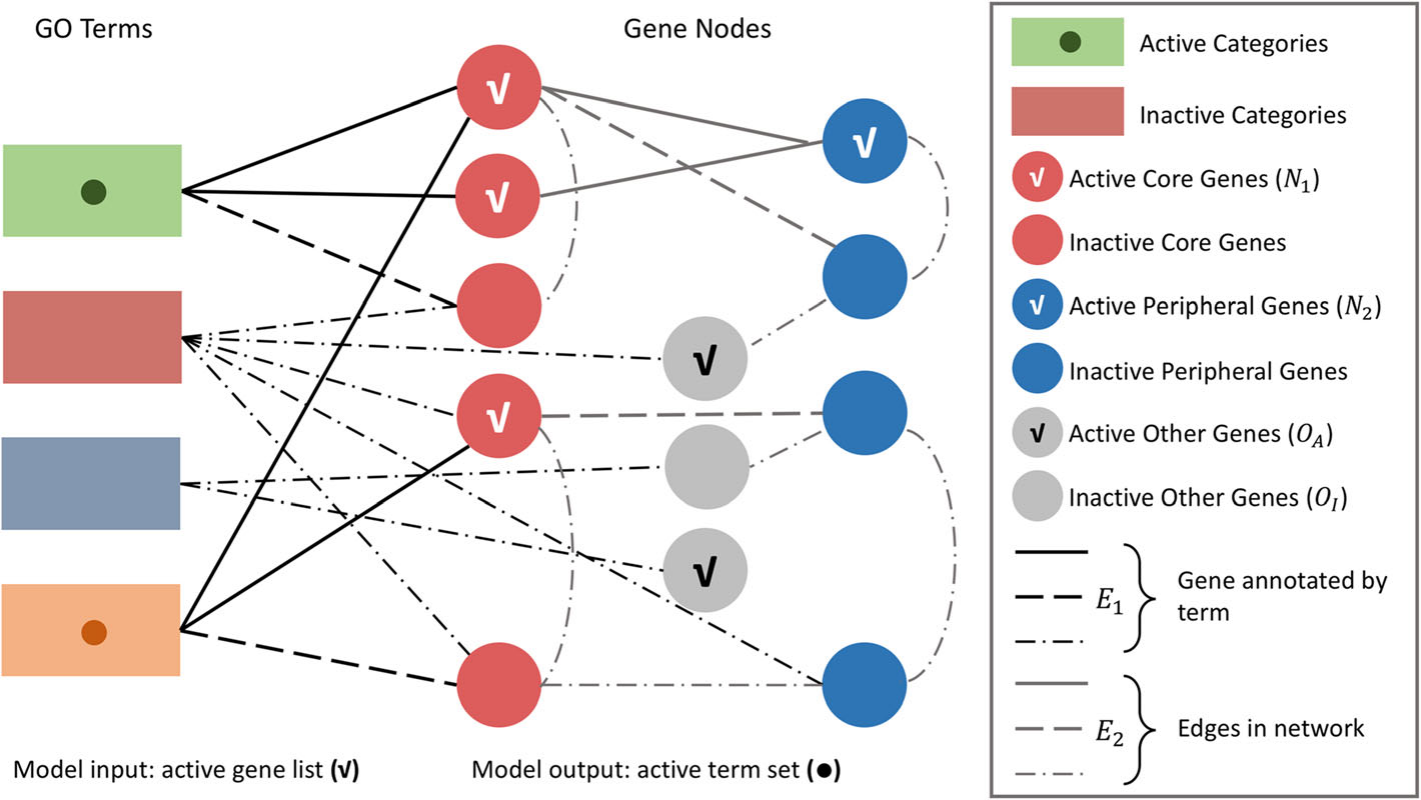
The tripartite graph for an intuitive interpretation of the generative process. Rectangular nodes represent the GO terms, and rounded nodes represent all genes annotated in one species. An edge is introduced between a GO term and a gene node, if and only if the gene is annotated by that term. The edges between two gene nodes are the counterpart edges in a biological network. The different line types represent different connection types: active-active, active-inactive, inactive-inactive

Intuitively, we can interpret this process in terms of a tripartite graph, which can represent the relationship between GO terms, core genes, peripheral genes and other genes on the biological network (Fig. 1). Given the set of active GO terms, the core genes (red nodes in Fig. 1) can be identified immediately based on GO annotation. According to the generative procedure mentioned above, by using the information of biological network, the peripheral genes, represented as blue nodes in Fig. 1, can also be found out. All the remaining genes are other genes, which are represented as gray nodes in Fig. 1. Three types of genes are selected into the active gene list by probabilities, *p*_1_, *p*_2_, *q*, respectively, where *p*_1_ > *p*_2_ ≫ *q*.

**Fig. 2:**
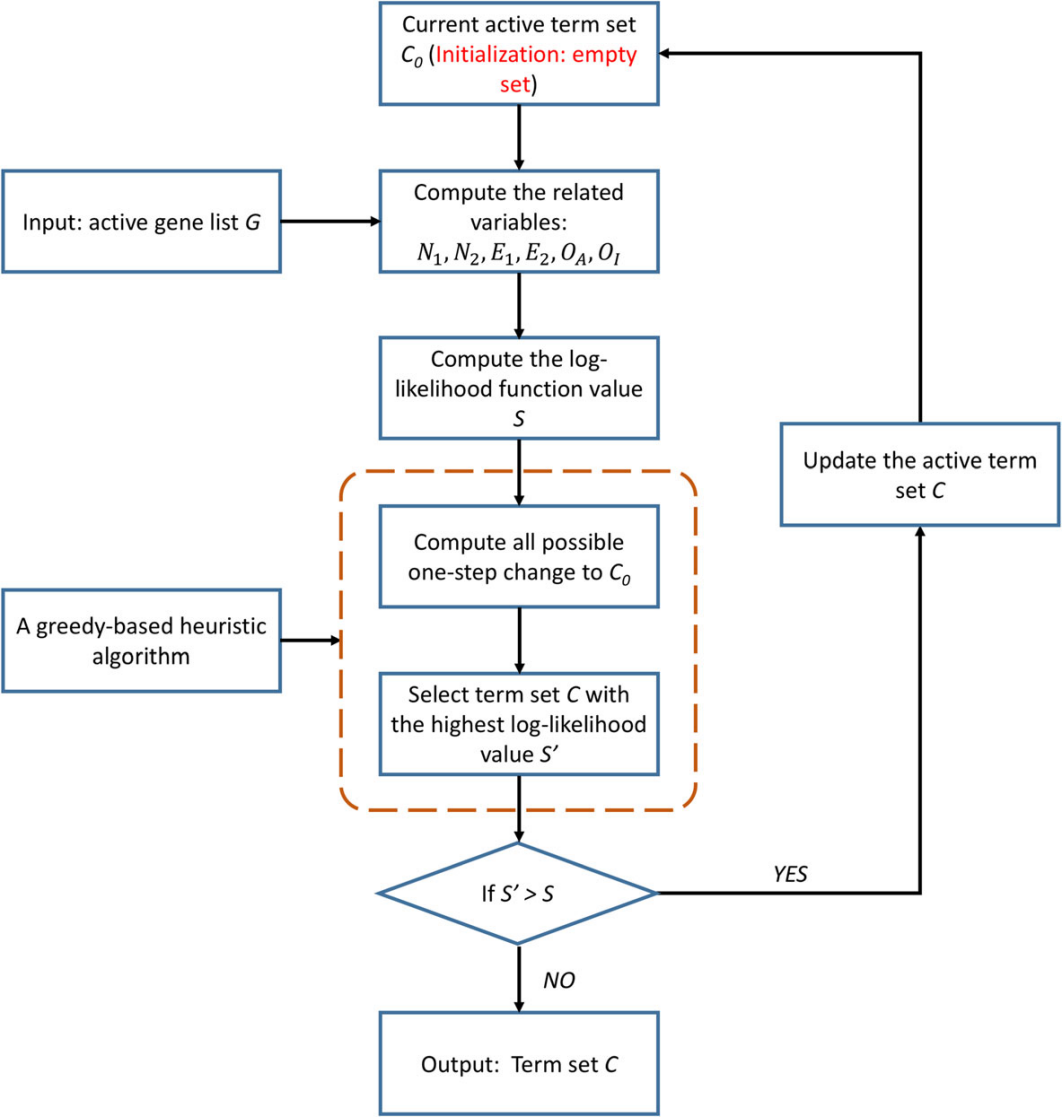
The workflow of NetGen. The active gene list *G* is the model input. We want to identify the most enriched GO term set *C*, which has a reasonable biological explanation to G, as the final output. A greedy-based heuristic algorithm was used to maximum the log-likelihood function

Note that though we all use the nomenclature “active” to describe the selected terms and genes, their underlying meanings are different. As shown in Fig. 1 and Fig. 2, the active genes are the model input whereas the active terms are the variables which need to be inferred as model output.

### Maximum likelihood estimation problem

Given an active gene list *G*, we infer the unknown active GO terms by maximizing the likelihood of observing the data. First, we give the following definitions as illustrated in Fig. 1:

(i) N_1_: Node set of active core genes.
(ii) * E*_*1*_: Edge set from active GO terms to inactive core genes.
(iii) N_2_: Node set of active peripheral genes.
(iv) * E*_*2*_: Edge set from core genes to inactive peripheral genes.
(v) * O*_*A*_: Node set of other active genes.
(vi) * O*_*I*_: Node set of other inactive genes.

Using these symbols, we defined the following log-likelihood function:

$$ L\left(G|C,{p}_1,{p}_2,q\right)=\left|{N}_1\right|\mathit{\log}{p}_1+\left|{E}_1\right|\mathit{\log}\left(1-{p}_1\right) $$

$$ \kern8.75em +\left|{N}_2\right|\mathit{\log}{p}_2+\left|{E}_2\right|\mathit{\log}\left(1-{p}_2\right)+\left|{O}_A\right|\mathit{\log}q+\left|{O}_I\right|\mathit{\log}\left(1-q\right)-\alpha \left|C\right| $$

where *C* denotes the set of inferred active GO terms and *G* is the observed active gene list. This log-likelihood function captures the generative property as we described above. First, the core genes had a relatively larger probability *p*_1_ to be active, and the peripheral genes had a relatively lower probability *p*_2_ to be active. Second, we penalized the inactive core genes and the inactive peripheral genes using the size of edge sets *E*_1_ and *E*_2_ instead of the size of node sets, respectively. In this way, we can reduce the redundancy of the active GO terms. Third, all the other genes both had the smallest probability *q* to be activated by external factors, such as noise, uncontrollable error in experiments, the incompletion of GO annotation and so on. In the end, a penalty term ∣*C*∣ was added in the likelihood function, so that the model will prefer a smaller set of active GO terms to facilitate the interpretation of the active gene list, and the parameter *α* was a positive number to balance the log-likelihood and the penalization term. The maximum likelihood estimation (MLE) approach was used for the functional enrichment analysis to identify the most enriched terms. In fact, maximizing the above log-likelihood function is equivalent to minimize the difference between the active gene list and the core and peripheral nodes activated by inferred active terms.

### Integer programming model and greedy algorithm

The above maximum likelihood estimation problem for searching the best GO term set *C* can be formulated as an integer quadratic programming (IQP) model. Supposing there are *M* GO terms and *N* genes, the annotations are denoted by an annotation matrix *A*, with dimension *M* × *N*. The element *A*_*ij*_ = 1, if the gene *j* is annotated by the term *i*, and *A*_*ij*_ = 0 otherwise. An active gene list is given by the vector *g* of length *N*. The element *g*_*i*_ = 1, if the gene *j* is in the active gene list, and *g*_*i*_ = 0 otherwise. The *N* × *N* matrix *B* is the adjacent matrix of biological network. The element *B*_*ij*_ = 1, if the gene *i* is connected to the gene *j* in network, and *B*_*ij*_ = 0 otherwise. The IQP model is formulated as follows:

$$ \underset{x,y,z}{\mathit{\max \limits }}\sum \limits_j{y}_j{g}_j\mathit{\log}{p}_1+\sum \limits_{i,j}{x}_i{A}_{ij}\left(1-{g}_j\right)\mathit{\log}\left(1-{p}_1\right)+\sum \limits_j{z}_j{g}_j\mathit{\log}{p}_2+\sum \limits_{i,j}{y}_i{B}_{i,j}\left(1-{y}_j\right)\left(1-{g}_j\right)\mathit{\log}\left(1-{p}_2\right)+\sum \limits_j\left(1-{y}_j\right)\left(1-{z}_j\right){g}_j\mathit{\log}q+\sum \limits_j\left(1-{y}_j\right)\left(1-{z}_j\right)\left(1-{g}_j\right)\mathit{\log}\left(1-q\right)-\alpha \sum \limits_i{x}_i $$

$$ {\displaystyle \begin{array}{ccc}s.t.& {y}_j\le \sum \limits_i{A}_{ij}{x}_i& j=1,2,\cdots, N\\ {}& {y}_j\ge {A}_{ij}{x}_i& i=1,2,\cdots, M;j=1,2,\cdots, N\\ {}& {z}_j\le \sum \limits_i{B}_{ij}{y}_i& j=1,2,\cdots, N\\ {}& {z}_j\ge {B}_{ij}{y}_i& i=1,2,\cdots, N;j=1,2,\cdots, N\\ {}& {x}_i\in \left\{0,1\right\}& i=1,2,\cdots, M\\ {}& {y}_j,{z}_j\in \left\{0,1\right\}& j=1,2,\cdots, N\end{array}} $$

The binary variable *x*_*i*_ denotes whether the term *i* is selected in the final output (i.e. active). *x*_*i*_ = 1 means that the term *i* is active, and *x*_*i*_ = 0 otherwise. The binary variable *y*_*j*_ denotes whether the gene *j* is a core gene, i.e. annotated by at least one active term. *y*_*j*_ = 1 if gene *j* is a core gene, and *y*_*j*_ = 0 otherwise. The binary variable *z*_*j*_ denotes whether the gene *j* is a peripheral gene. *z*_*j*_ = 1 if gene *j* is a peripheral gene, and *z*_*j*_ = 0 otherwise. Using these symbols, the sizes of *N*_1_ , *E*_1_ , *N*_2_ , *E*_2_ , *O*_*A*_ , *O*_*I*_ defined in MLE can be calculated as the corresponding items in the objective function of IQP. Besides, the constraints imply the basic assumptions in our generative model. ${y}_j\le \sum \limits_i{A}_{ij}{x}_i$ and *y*_*j*_ ≥ *A*_*ij*_ *x*_*i*_ restrict that only the genes linked with at least one active term can be a core gene. ${z}_j\le \sum \limits_i{B}_{ij}{y}_i$ and *z*_*j*_ ≥ *B*_*ij*_ *y*_*i*_ restrict that only the genes linked with at least one core gene in network can be a peripheral gene.

The above IQP is difficult to solve exactly since integer programming generally is NP-hard, from the perspective of computational complexity. It may not be applicable for real annotation data analysis, which make us turn to use a heuristic algorithm to seek for an approximate solution. As in GenGO [19], we used a greedy algorithm to find the near optimal set of GO terms. Briefly, the algorithm first finds out a single term whose log-likelihood function value is the highest. Then in each iteration, the algorithm considers all possible one-step changes of the current set of active terms, i.e. adding or deleting one term each time. It records the term that make the largest improvement to the current log-likelihood function value, and consequently updates the current term set. The algorithm stops if the log-likelihood function value cannot be further improved by any one-step change.

In conclusion, the whole workflow of NetGen is shown in Fig. 2. First, using the active gene list *G* and the current active term set *C*_0_, determine the core and peripheral genes and compute the related numbers *N*_1_ , *N*_2_ , *E*_1_ , *E*_2_ , *O*_*A*_ , *O*_*I*_. Second, compute the log-likelihood function value *S* of the current active term set *C*_0_. Third, using a greedy-based heuristic algorithm to obtain an alternative updated active term set *C*. Last, update the current term set *C*_0_ iteratively until no improvement could be achieved by any one-step change and then output the final term set.

### Mixed parameter selection strategy

The selection of model parameters is important and difficult. When applying NetGen in practical applications, in fact, the true parameters for generating the active gene list is largely unknown, and the inappropriate parameter selection may affect the performance of enrichment analysis. To alleviate the effects of parameter selection, parameter sensitivity analysis (see Additional file 1) was performed first to test the robustness of NetGen parameters. According to the helpful information supplied from the sensitivity analysis, we designed a mixed parameter selection strategy to facilitate the use of NetGen in real data analysis.

Given a list of active genes, the following mixed parameter selection strategy was performed to produce multiple solutions, which can offer more information to the biologist for downstream analysis.

1. The candidate values for model parameters were fixed as *p*_1_ = 0.8 or 0.5, *p*_2_ = 0.1 or 0.05, *q* = 0.01 or 0.001, *α* = 3.
2. Run NetGen algorithm using all eight combinations of candidate parameter values. An active term set was obtained for each parameter combination.
3. Union the genes annotated by at least one term in the active term set to form a super pseudo term for each parameter combination.
4. For each super pseudo term, compute the enrichment *p*-value using the Fisher’s exact test.
5. Output the results of all eight parameter combinations in ascending order of *p*-values.

As for GenGO, the model is unrelated to the parameter *p*_2_. All four parameter combinations (*p*_1_ = 0.8 or 0.5, *q* = 0.01 or 0.001, *α* = 3) were used to perform the same mixed parameter selection strategy. Ultimately, the results of four parameter combinations are output in ascending order of *p*-values.

### Simulated datasets

In our study, we first tested the effectiveness of NetGen via simulation studies, on the biological process (BP), the molecular function (MF) and the cellular component (CC) domains, respectively. Two other alternative methods, GenGO [19] and Fisher’s exact test [10], were also taken into consideration for comparison. Here, we used the following four groups of simulation parameters (generating parameters) to generate the related active gene list:

1. *p*_1_ = 0.8,*p*_2_ = 0.3,*q* = 0.001, *α* = 3
2. *p*_1_ = 0.5,*p*_2_ = 0.3,*q* = 0.001, *α* = 3
3. *p*_1_ = 0.8,*p*_2_ = 0.1,*q* = 0.001, *α* = 3
4. *p*_1_ = 0.8,*p*_2_ = 0.3,*q* = 0.01, *α* = 3

The whole workflow of simulation studies is as follows:

1. We restricted the terms in one domain (BP, CC or MF), whose number of covered gene was 2 to 500 (to remove the terms too specific or too general), and then randomly selected 500 terms 10 times from this refined term set to obtain 10 annotation sets.
2. In each annotation set, we randomly selected 5 terms 20 times as the target active term set. For each target active term set, we generated the active gene list using a fixed generating parameter combination.
3. Each active gene list was used as the model input. The solving parameter values of NetGen were the same as the generating parameter values. Since GenGO is unrelated to parameter *p*_2_, we only used the values of *p*_1_ , *q* , *α* to obtain the output terms.
4. The 200 model outputs were combined to obtain a 2 × 2 contingency table. Besides, the Bonferroni corrected hypergeometric test *p*-values were used as the significant scores for these output terms.
5. The precision-recall curves were plotted to test the performance of each method.

For each generating parameter combination, we plot a precision-recall curve, on which each point corresponds to a cutoff of corrected hypergeometric test *p*-value. The precision and recall are defined as:

$$ \mathrm{precision}=\frac{TP}{TP+ FP} $$

$$ \mathrm{recall}=\frac{TP}{TP+ FN} $$

where *TP*, *FP* and *FN* are the abbreviations for true positive, false positive and false negative. *TP* is the number of true active terms below the cutoff. *FP* stands for the number of inactive terms below the cutoff. *FN* is the number of active terms above the cutoff. We set the significant scores of terms that are not in the model output to 1, for ensuring the correct calculation of recall.

### Real datasets

In our study, we used two kinds of data, GO annotation and PPI network, to identify the active terms via NetGen.

The GO annotation was extracted from R package *org.Hs.eg* *.db* in Bioconductor project. The detailed information about the GO annotation data was summarized in Additional file 1 Table S4.

PPI data was extracted from Human Protein Reference Database (HPRD, http://www.hprd.org/) [23]. After removing the multiple edges and the self-loops, the refined PPI network contained 9453 genes and 36,867 interactions. In our model, we did not restrict our analysis on the overlapped genes (i.e. genes included in the PPI network and annotated by at least one GO term). Instead, we used the network information of the overlapped core genes to assist our model to identify the enriched terms.

To test the performance of NetGen in real data applications, four microarray gene expression datasets of human complex diseases were selected from the Gene Expression Omnibus repository (accession number GSE4115, GSE11223, GSE9750, GSE36895, respectively), basing on several criteria (see Additional file 1). After the preprocessing of the original datasets, we sorted the microarray genes by ascending order of the *p*-values derived by the Student’s t-test on the disease and control samples. The top 100 genes were selected as the differential expression gene set. The differential expression gene set was then overlapped with the annotated genes, which were used as the final active gene list to perform the enrichment analysis.

### Semantic similarity based analysis

The GO semantic similarity has been widely used in the field of bioinformatics. It provides a criterion to measure the redundancy between the functional terms. Generally speaking, a lower semantic similarity score indicates a lower redundancy between two GO terms. In this paper, we used the averaged GO semantic similarity score to measure the redundancy of the identified terms. The averaged GO semantic similarity score is defined as:

$$ ASS(S)={\left(\genfrac{}{}{0pt}{}{n}{2}\right)}^{-1}\sum \limits_{1\le i<j\le n} score\left({S}_i,{S}_j\right) $$

where *S* = {*S*_1_, *S*_2_,  ⋯ , *S*_*n*_} is the identified term set and *n* is the size of *S*. *score*(*S*_*i*_, *S*_*j*_) is the semantic similarity score between GO terms *S*_*i*_ and *S*_*j*_. To make the result more comparable, a background distribution of the averaged semantic similarity scores was derived on each dataset, which was obtained by randomly resampling term sets with same size for 100,000 times. As for the Fisher’s exact test, the most enriched top *n* terms were selected. *n* is the term set size identified via NetGen. In this work, the semantic similarity score was computed using the R package GOSemSim [24], which is compiled in Bioconductor [25].

## Results

### Simulation studies

**Fig. 3:**
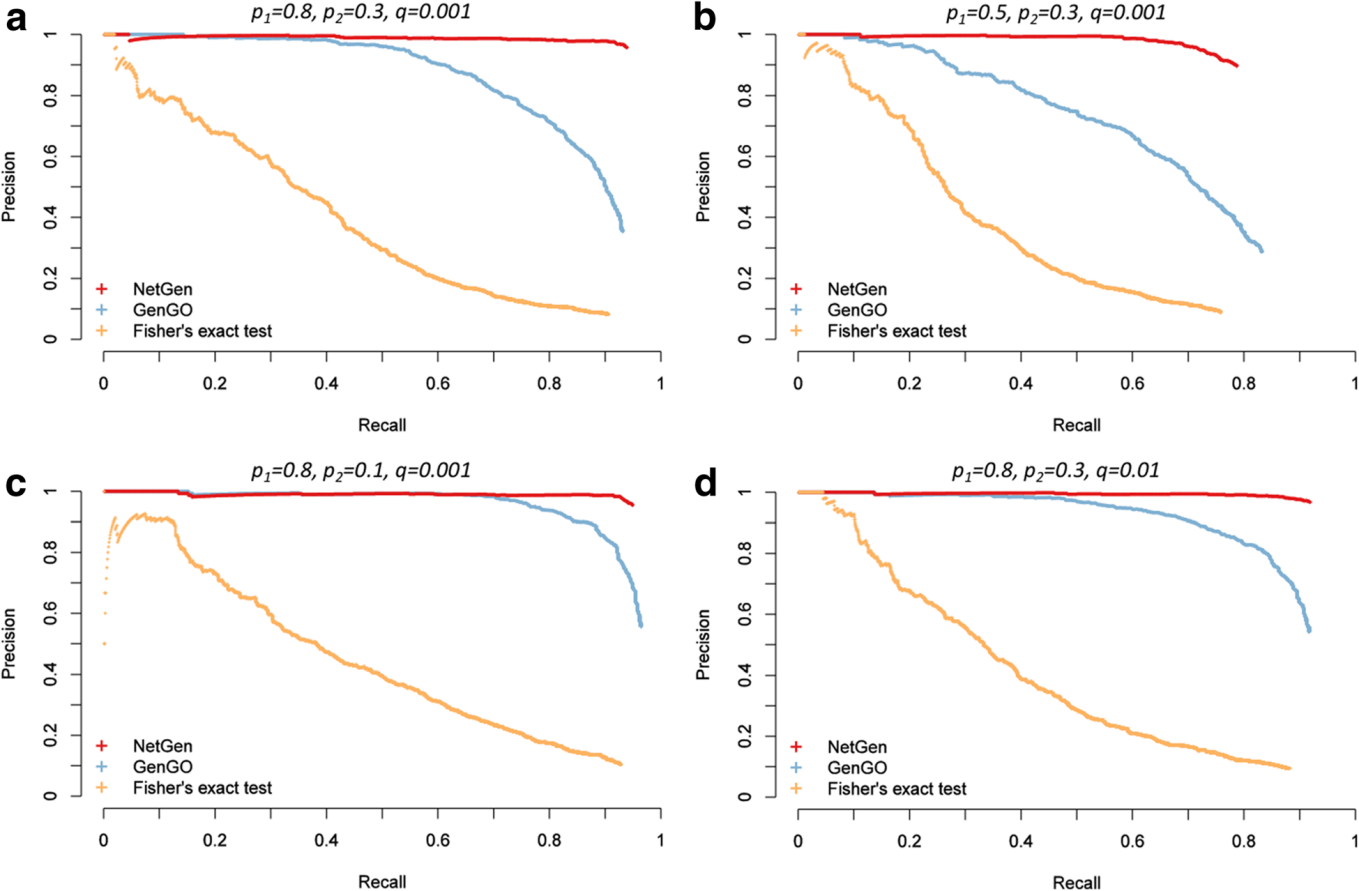
The performance of NetGen and alternative methods on biological process (BP) domain. Each panel stands for a setting of generating parameters. The performance of NetGen, GenGO and Fisher’s exact test are shown in red, blue and orange respectively. The active gene lists were simulated under the assumption of NetGen

We first test the performance of NetGen via simulation studies. The detailed description of our simulation study can be found in Methods. The results on biological process, cellular component and molecular function domain are shown in Fig. 3, Additional file 1 Fig. S5 and S6, respectively.

From the results, we can see that NetGen outperformed other alternative methods on both three domains, and showed a more stable performance when using all kinds of parameter combinations. Fisher’s exact test is independent of the term combinations and neglects the overlap of parent or descendant terms. Consequently, an inferior performance was observed since highly redundant terms were identified. The performance of GenGO was closely related to the selection of generating parameter *p*_1_ and *p*_2_, when the true active gene list is explicable using network information. In detail, the performance of GenGO significant decreased, when the impact of network becomes larger, i.e. *p*_2_/*p*_1_ increased (Fig. 3, Fig. S5-S6 B). A comparable performance was observed, if the original active gene list was less impact by the network information, i.e. *p*_2_/*p*_1_ decreased (Fig. 3, Fig. S5-S6 C). Besides, increasing probability *q*, the influence of noise or other uncontrollable error in experiment, can improve the performance of GenGO (Fig. 3, Fig. S5-S6 D), which was accordance with the sensitivity analyses of *q* (see Fig. S3). This may be explained by that the noise itself can offset the effect of active genes generated via network propagation.

In addition to the above simulation procedure, we also simulated another alternative circumstance. We wonder how the performance of NetGen behaved, if the active gene lists were actually unrelated to the network information. Therefore, active gene lists were generated under the assumption of GenGO model, i.e. *p*_2_ = 0. The performance and discussion can be found in Additional file 1 (Figure S7-S9), which showed that NetGen with small *p*_2_ can successfully handle both cases of simulated datasets generated by GenGO model and NetGen model.

### Real data studies

To test the efficiency of NetGen in real datasets, we used four GEO microarray gene expression profiles of human complex diseases to execute real data analysis. More details about the data description and the preprocessing can be found in Additional file 1.

For each dataset, the dataset-specific active gene list was generated as introduced in the Methods section. Since the true parameter combination for generating active gene list from real datasets is unknown, we performed a mixed parameter selection strategy to obtain multiple solutions (see Methods). The evaluation of the mixed parameter selection strategy on the simulation studies showed that the generating parameters are not necessarily the best solving parameters (see Additional file 1 Figure S10-S11). Instead to infer the true values of the generative parameters, which is very difficult and may be meaningless, the mixed parameter selection strategy intends to produce multiple solutions in real applications, and reveal more information of the underlying biological processes for the downstream analysis. Due to the space limit, we only analyzed the enriched term set with the lowest combination *p*-value. The results of enrichment analysis are shown in Table 1, 2, 3 and 4. The GO terms that were not identified by GenGO with same mixed parameter selection strategy are shown in bold.

**Table 1:** The enrichment analysis result of NetGen on lung cancer dataset

Rank	GO ID	Description	*p*-value
1	GO:0006491	N-glycan processing	1.32e-3
2	GO:0006662	glycerol ether metabolic process	1.93e-3
3	GO:0006175	dATP biosynthetic process	5.54e-3
4	GO:0060149	negative regulation of posttranscriptional gene silencing	5.54e-3
5	GO:0006043	glucosamine catabolic process	5.54e-3
6	GO:0035772	interleukin-13-mediated signaling pathway	5.54e-3
7	**GO:2000832**	negative regulation of steroid hormone secretion	1.11e-2
8	GO:0021648	vestibulocochlear nerve morphogenesis	1.11e-2
9	GO:0072318	clathrin coat disassembly	1.11e-2
10	GO:1900748	positive regulation of vascular endothelial growth factor signaling pathway	1.65e-2
11	GO:0015014	heparan sulfate proteoglycan biosynthetic process, polysaccharide chain biosynthetic process	1.65e-2
12	GO:0000710	meiotic mismatch repair	1.65e-2
13	GO:0030070	insulin processing	1.65e-2
14	**GO:0072190**	ureter urothelium development	1.65e-2
15	GO:0034154	toll-like receptor 7 signaling pathway	2.20e-2
16	**GO:2000195**	negative regulation of female gonad development	1
17	**GO:0016082**	synaptic vesicle priming	1
18	**GO:2000370**	positive regulation of clathrin-dependent endocytosis	1
19	**GO:0002143**	tRNA wobble position uridine thiolation	1
20	**GO:0060978**	angiogenesis involved in coronary vascular morphogenesis	1

Best parameter setting: *p*_1_=0.5, *p*_2_=0.05, *q*=0.001. Term combination *p*=1.50e-24

An appropriate parameter combination identified via a mixed parameter selection strategy was shown at the bottom of the table. The Fisher’s exact test *p*-values for single term and term combination were listed. The GO terms in bold were particularly identified by NetGen

As for lung cancer dataset, we obtained a significantly enriched term set, whose combination *p*-value computed by Fisher’s exact test was 1.5 × 10^−24^, including 20 lung-related terms (Table 1). Notably, five terms (GO:2000195, GO:0016082, GO:2000370, GO:0002143, GO:0060978) with *p*-value equal to 1 were particularly identified by NetGen, i.e. these terms are not directly annotated to the active genes. However, these five terms discovered when taking the supplementary role of network information into consideration actually showed a closely relationship with lung tumorigenesis. For examples, mutations in clathrin and several of its associated proteins and adaptors (EPS15, HIP1, CALM, endophilin and β-arrestin 1) were identified via systematic characterization of somatic mutations in breast, renal and lung cancers [26, 27], which is the main function in term GO:2000370 (positive regulation of clathrin-dependent endocytosis). As for GO:0016082 (synaptic vesicle priming), exosome is closely related to synaptic vesicle cycle. In pathological states, such as cancer, a number of key proteins and microRNAs are expulsed due to the exosome-mediated abnormal activity of the export machinery [28, 29]. For GO:0002143 (tRNA wobble position uridine thiolation), the aberrant expression of tRNA modification plays an important role in complex diseases [30]. Besides, thiolation-based chemotherapy has been proposed for lung cancer [31].

The combination *p*-value of identified term set for ulcerative colitis dataset was 1.33 × 10^−27^, including 18 disease-related terms (Table 2). Adding the network information assisted our model to identify three terms (GO:1901299, GO:1901841, GO:0043547, Fisher’s exact test *p*-value = 1), which have no directly connections with the active genes. These three terms showed closely relationships with carcinogenesis. For example, as for term GO:1901841 (regulation of high voltage-gated calcium channel activity), substantial researches showed that calcium channel intimately connected with cancer cells proliferation and metastasis [32, 33]. For GO:1901299 (negative regulation of hydrogen peroxide-mediated programmed cell death), hydrogen peroxide (H_2_O_2_) plays a key role in tumorigenesis. Superfluous increasing of H_2_O_2_ generated by cancer cell may lead to several pivotal changes, such as DNA alteration, cell proliferation, apoptosis and angiogenesis, during tumorigenesis [34, 35].

**Table 2:** The enrichment analysis result of NetGen on ulcerative colitis dataset

Rank	GO ID	Description	*p*-value
1	**GO:0032968**	positive regulation of transcription elongation from RNA polymerase II promoter	1.10e-3
2	GO:0018874	benzoate metabolic process	3.83e-3
3	GO:0010900	negative regulation of phosphatidylcholine catabolic process	3.83e-3
4	GO:1900402	regulation of carbohydrate metabolic process by regulation of transcription from RNA polymerase II promoter	3.83e-3
5	GO:0006294	nucleotide-excision repair, preincision complex assembly	3.83e-3
6	GO:0038193	thromboxane A2 signaling pathway	3.83e-3
7	GO:0031119	tRNA pseudouridine synthesis	7.65e-3
8	GO:0007439	ectodermal digestive tract development	7.65e-3
9	GO:0009240	isopentenyl diphosphate biosynthetic process	1.15e-2
10	GO:0045196	establishment or maintenance of neuroblast polarity	1.15e-2
11	GO:0006154	adenosine catabolic process	1.15e-2
12	GO:0002254	kinin cascade	1.15e-2
13	GO:2000681	negative regulation of rubidium ion transport	1.15e-2
14	**GO:0035701**	hematopoietic stem cell migration	1.52e-2
15	GO:0008612	peptidyl-lysine modification to peptidyl-hypusine	1.52e-2
16	**GO:1901299**	negative regulation of hydrogen peroxide-mediated programmed cell death	1
17	**GO:1901841**	regulation of high voltage-gated calcium channel activity	1
18	**GO:0043547** ^**a**^	positive regulation of GTPase activity	1
Best parameter setting: *p*_1_=0.5, *p*_2_=0.05, *q*=0.001.		Term combination *p*=1.33e-27	

^a^GO:0032850 updated to alternate term GO:0043547

An appropriate parameter combination identified via a mixed parameter selection strategy was shown at the bottom of the table. The Fisher’s exact test *p*-values for single term and term combination were listed. The GO terms in bold were particularly identified by NetGen

For cervical carcinogenesis dataset, a set of 23 disease-related terms, whose combination *p*-value was 1.10 × 10^−37^, were identified (Table 3). Among these identified terms, four terms (GO:0000741, GO:2000656, GO:0032848, GO:0090158), which had no directly overlap with the active genes, were recovered when adding the network information. Particularly, GO:2000656 is related to the regulation of apolipoprotein binding. Many studies showed that apolipoprotein took part in the tumor progression [36, 37]. The function of GO:0032848 (negative regulation of cellular pH reduction) is mainly connected with the regulation of intracellular acid-base. The particular mechanisms of pH sensing and regulation in tumor can be a common physical hallmark of solid tumors [38].

**Table 3:** The enrichment analysis result of NetGen on cervical carcinogenesis dataset

Rank	GO ID	Description	*p*-value
1	GO:0006271	DNA strand elongation involved in DNA replication	3.42e-11
2	**GO:0090224**	regulation of spindle organization	1.78e-3
3	GO:0001927	exocyst assembly	6.43e-3
4	GO:0038016	insulin receptor internalization	6.43e-3
5	**GO:0070676**	intralumenal vesicle formation	6.43e-3
6	GO:0086042	cardiac muscle cell-cardiac muscle cell adhesion	6.43e-3
7	**GO:0014738**	regulation of muscle hyperplasia	1.28e-2
8	GO:2000393	negative regulation of lamellipodium morphogenesis	1.28e-2
9	**GO:0010993**	regulation of ubiquitin homeostasis	1.28e-2
10	GO:0006050	mannosamine metabolic process	1.28e-2
11	**GO:0046602**	regulation of mitotic centrosome separation	1.92e-2
12	GO:0072708	response to sorbitol	1.92e-2
13	GO:0001992	regulation of systemic arterial blood pressure by vasopressin	1.92e-2
14	GO:1902498	regulation of protein autoubiquitination	1.92e-2
15	GO:0048388	endosomal lumen acidification	1.92e-2
16	**GO:0048280**	vesicle fusion with Golgi apparatus	2.55e-2
17	GO:0097264	self proteolysis	3.18e-2
18	GO:0045329	carnitine biosynthetic process	3.18e-2
19	**GO:0051382**	kinetochore assembly	7.45e-2
20	**GO:0000741**	karyogamy	1
21	**GO:2000656**	regulation of apolipoprotein binding	1
22	**GO:0032848**	negative regulation of cellular pH reduction	1
23	**GO:0090158**	endoplasmic reticulum membrane organization	1

Best parameter setting: *p*_1_=0.5, *p*_2_=0.05, *q*=0.001. Term combination *p*=1.10e-37

An appropriate parameter combination identified via a mixed parameter selection strategy was shown at the bottom of the table. The Fisher’s exact test *p*-values for single term and term combination were listed. The GO terms in bold were particularly identified by NetGen

As for renal cell carcinoma dataset, the combination *p*-value of identified term set, including 27 terms, was 8.68 × 10^−50^ (Table 4). Similar phenomena were also observed for the *p*-values of three terms (GO:0002542, GO:0014858 and GO:0010766) were equal to 1, which revealed the function of network information. For example, the function of GO:0010766 (negative regulation of sodium ion transport) is related to the transportation of sodium ion. The control of the ionic equilibrium is the major function of kidney [39]. The similar term was also identified by *Tun HW* et al., when using the different expression profiles of clear cell renal cell carcinoma [40]. For GO:0002542 (Factor XII activation), the activity of Factor XII is closely connected with the phenomenon, known as the enhanced permeability and retention (EPR) effect, which has been observed to be universal in solid tumors for lipid and macromolecular agents [41].

**Table 4:** The enrichment analysis result of NetGen on renal cell carcinoma dataset

Rank	GO ID	Description	*p*-value
1	GO:0090259	regulation of retinal ganglion cell axon guidance	7.56e-7
2	**GO:0033572**	transferrin transport	1.05e-6
3	GO:0072017	distal tubule development	3.03e-5
4	GO:2000054	negative regulation of Wnt signaling pathway involved in dorsal/ventral axis specification	3.34e-5
5	**GO:0072015**	glomerular visceral epithelial cell development	1.17e-3
6	GO:2000287	positive regulation of myotome development	5.81e-3
7	GO:0006113	fermentation	5.81e-3
8	GO:0051460	negative regulation of corticotropin secretion	5.81e-3
9	GO:0060720	spongiotrophoblast cell proliferation	5.81e-3
10	GO:0043438	acetoacetic acid metabolic process	5.81e-3
11	GO:0032972	regulation of muscle filament sliding speed	5.81e-3
12	**GO:0090038**	negative regulation of protein kinase C signaling	1.16e-2
13	GO:0035425	autocrine signaling	1.16e-2
14	GO:0010760	negative regulation of macrophage chemotaxis	1.16e-2
15	**GO:0060161**	positive regulation of dopamine receptor signaling pathway	1.73e-2
16	GO:0097411	hypoxia-inducible factor-1alpha signaling pathway	1.73e-2
17	GO:0060435	bronchiole development	1.73e-2
18	**GO:0051933**	amino acid neurotransmitter reuptake	1.73e-2
19	GO:0046598	positive regulation of viral entry into host cell	2.31e-2
20	GO:0015015	heparan sulfate proteoglycan biosynthetic process, enzymatic modification	2.87e-2
21	GO:0006572	tyrosine catabolic process	2.87e-2
22	GO:0019532	oxalate transport	2.87e-2
23	**GO:0072171**	mesonephric tubule morphogenesis	3.44e-2
24	**GO:0051156**	glucose 6-phosphate metabolic process	5.12e-2
25	**GO:0002542**	Factor XII activation	1
26	**GO:0010766**	negative regulation of sodium ion transport	1
27	**GO:0014858**	positive regulation of skeletal muscle cell proliferation	1

Best parameter setting: *p*_1_=0.5, *p*_2_=0.05, *q*=0.001. Term combination *p*=8.68e-50

An appropriate parameter combination identified via a mixed parameter selection strategy was shown at the bottom of the table. The Fisher’s exact test *p*-values for single term and term combination were listed. The GO terms in bold were particularly identified by NetGen

**Fig. 4:**
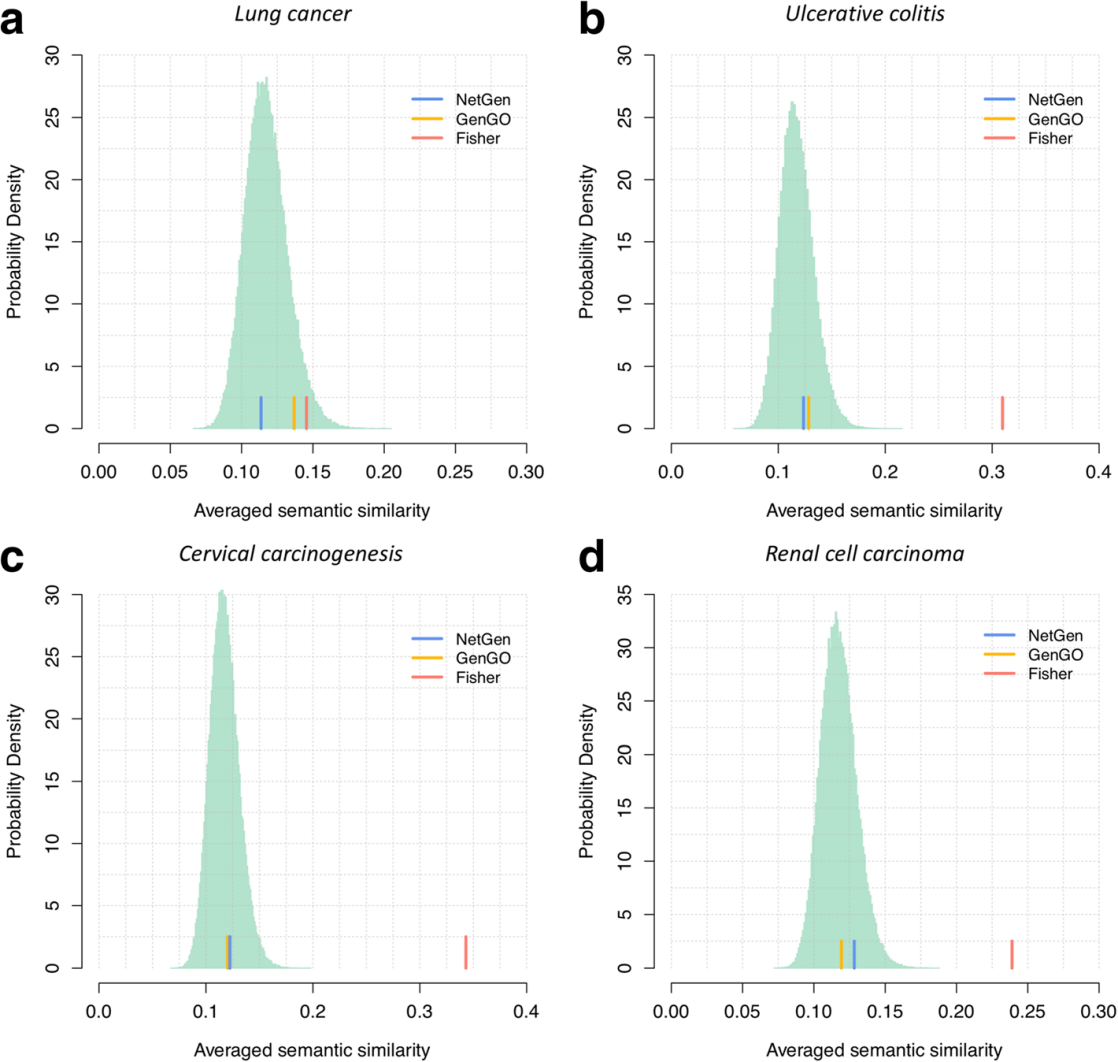
Comparison of the averaged semantic similarity score in the identified term set. The light green distribution represents the semantic similarity score at the random level. The blue, orange and red bar represent the NetGen, GenGO and Fisher’s exact test, respectively. The semantic similarity score was computed using the GOSemSim package [24] in R

The identified enriched terms with a higher similarity and redundancy often makes the researchers harder to obtain the underlying biological interpretations. In addition to the above enriched analysis, we test the redundancy of the enriched terms identified via NetGen. Here, the averaged semantic similarity score was used to measure the redundancy of the identified terms (see Methods). The results can be found in Figure 4, which showed that the averaged semantic similarity scores of NetGen and GenGO, two combination-based approaches, were far below than the scores of the Fisher’s exact test on four datasets. Besides, the score of NetGen was around the mean score of the random distribution, which indicates the redundancy of these identified terms were nearly minimized. In conclusion, NetGen can effectively reduce the redundancy of the identified terms, which is helpful in the exploration of the underlying pathogenesis of complex diseases.

## Discussion

The innovative component of NetGen is the integration of network information to extend the generative model of functional enrichment analysis. The framework of NetGen enables the users to exploit not only the PPI network but also various other distinct biomolecular networks, such as gene regulatory network, metabolic network and signal transduction network. Since different types of biomolecular networks reveal different levels and essential biological mechanisms in biological system, the selection of biomolecular network does have an influence on the performance of NetGen. Here, we did not compare the performance of NetGen with different biomolecular networks. One can select an appropriate network to assist the functional enrichment analysis according to the studied biological problem and available datasets. Another important point is the completeness or quality of the used network. The performance of NetGen would be greatly affected by the highly-noisy biological network since the wrong peripheral nodes due to noisy edges in the network will mess the results of enrichment analysis. Relatively speaking, the influence of the incomplete network may be small because the NetGen model will degenerate to some extent into the GenGO model. According to the parameter sensitivity analysis (see Additional file 1), it is better to choose a smaller *p*_2_ when using a network with low quality.

The generative model is a computational model that assumes that the observed data was generated under certain probabilistic model and some distributions. The observed data was then used to estimate the parameters of the probabilistic model and distributions, and to infer values that could not be directly observed. Through maximizing a log-likelihood function, NetGen can identify the most likely significant terms. In this paper, we used a greedy approximation algorithm to seek for a near-optimal solution of the 0–1 integer programming problem. Based on this greedy algorithm, the running time of NetGen, depending on the size of input annotation matrix, is acceptable for large datasets in real applications. On the other hand, the solution quality of functional enrichment analysis is also affected by the approximation algorithm. In this work, the performance of NetGen using different approximation algorithm was not compared, which will be one of the goals in our future research.

There are three main parameters, *p*_1_ , *p*_2_ , *q*, in our model, which were explained in the generative process. We executed the parameter sensitivity analysis (see Additional file 1) to test their robustness in related enrichment analysis. The sensitivity analysis result can be a reference to help the user selecting an appropriate parameter combination. Besides, one can obtain a more explicit and intuitive explanation from some special cases. If the parameters are set as *p*_1_ = 1 , *p*_2_ = 0 , *q* = 0, the model is equivalent to identify the term set that the union of their directly annotated genes has the most overlap with the active gene list. Similarly, if the parameters are set as *p*_1_ = 1 , *p*_2_ = 1 , *q* = 0, the annotated genes in one term should add the corresponding neighbor genes in biological network. From this perspective, NetGen is a generalization to the above simple enrichment strategy and unifies these model into one framework.

Usually the biologists will obtain one ranked gene list for functional enrichment analysis. For example, the differential expression genes are often ranked by the t-test *p*-value. The rank information of gene list is not exploited in NetGen. It will be useful if the rank information of gene list can be considered and the outputs of functional enrichment analysis may become more precise. However, it is very difficult to integrate the rank information into the current term combination-based approaches. To the best of our knowledge, such kind of methods has not been studied in literature (see Additional file 1 Table S4). This will be one of the directions in our future research.

## Conclusions

In this paper, a novel network-based probabilistic generative model, NetGen, was proposed to perform the enrichment analysis. An additional protein-protein interaction network was explicitly used to assist the functional enrichment analysis. NetGen achieved a superior performance than other compared methods in the simulation studies. Besides, several important GO terms, which were not directly linked with the active gene list, were exclusively identified by NetGen on real datasets.

In real applications, NetGen was not restrict on *Homo sapiens* but can also be applied on any other species. Our procedure leads to a more reasonable and explainable result of the functional enrichment analysis. As a novel term combination-based functional enrichment analysis method, NetGen is complementary to current individual term-based methods. We believed that NetGen is an efficient computational tool for functional enrichment analysis and can help to explore the underlying pathogenesis of complex diseases.

## Additional file

Additional file 1: Supplementary materials including the classification of enrichment analysis methods, the parameter sensitivity analysis, the additional simulation results, and the description of gene expression datasets, GO annotation data, and active gene lists used in real data applications. (PDF 1385 kb)

## Abbreviations

BP: Biological process
CC: Cellular component
FN: False negative
FP: False positive
GEO: Gene Expression Omnibus
GO: Gene ontology
IQP: Integer quadratic programming
MF: Molecular function
MLE: Maximum likelihood estimation
PPI: Protein-protein interaction
TP: True positive
