# Supplementary material for: NetGen: a novel network-based probabilistic generative model for gene set functional enrichment analysis
Source: BMC Syst Biol. 2017 Sep 21;11(Suppl 4):75. doi: 10.1186/s12918-017-0456-7 (PMC5615262; doi:10.1186/s12918-017-0456-7)
Supplement: Supplementary file 1 — Supplementary materials including the classification of enrichment analysis methods, the parameter sensitivity analysis, the additional simulation results, and the description of gene expression datasets, GO annotation data, and active gene lists used in real data applications. (PDF 1385 kb) [file 12918_2017_456_MOESM1_ESM.pdf]

Supplementary Materials

NetGen: a novel network-based probabilistic generative model for gene set functional enrichment analysis

Duanchen Sun[1,2,3], Yinliang Liu[1,2,3], Xiang-Sun Zhang[1], Ling-Yun Wu[1,2,3,*]

[1]Institute of Applied Mathematics, Academy of Mathematics and Systems Science, Chinese Academy of Sciences, Beijing 100190, China

[2]National Center for Mathematics and Interdisciplinary Sciences, Chinese Academy of Sciences, Beijing 100190, China

[3]University of Chinese Academy of Sciences, Beijing 100049, China

*To whom correspondence should be addressed. Email: lywu@amss.ac.cn

1. Classification of different methods for enrichment analysis

Basing on the model input (type of gene list: gene set only or full gene list with score) and the output (evaluation pattern of the identified GO terms: single term or term combination), the current functional enrichment analysis methods can be briefly categorized into three classes (Table S1, Class I-III).

Table S1. Classification of different methods for enrichment analysis.

Input \ Output	Single Term	Term Combination
Gene set only	Class I	Class III
All genes with score	Class II	Class IV*

*Not found in literature.

Actually, one could model a particular type of method that uses all genes with scores as model input and outputs one or more term combinations (Table S1, Class IV). However, such kind of method has not been studied in literature, to the best of our knowledge.

2. Parameter sensitivity analysis

2.1 Workflow

Based on the original assumption of our network-based generative model, we have $p_1 > p_2 \gg q$. However, the true parameter combination for generating active gene list is largely unknown, and may be related to the studied biological problem. The inappropriate selection of solving parameters may affect the performance of enrichment analysis. Therefore, a parameter sensitivity analysis, performed on biological process (BP) domain, was executed first to test the robustness of each model parameter.

For each model parameter combination (p_1, p_2, q) in sensitivity analysis, we used a preset generating values to simulate an active gene list, then a wide-range solving parameter values were used to test the robustness of method. The preset generating and solving values for each model parameter are shown in Table S2.

Table S2: The summary of model parameter values selection in our sensitivity analysis.

Model parameter	Generating values	Solving values
p_1	0.8, 0.5	0.1, 0.3, 0.5, 0.7, 0.9
p_1	0.3, 0.1	0.1, 0.3, 0.5, 0.7, 0.9
q	0.001, 0.01	0.001, 0.005, 0.01, 0.05, 0.1, 0.2
α		0.1, 1, 3, 10, 100, 1000

In our generative model, parameter q stands for the influence of noise and uncontrollable error in experiment. Since the number of human genes is four orders of magnitude, we first chose $q = 0.001$ by experience, which resulted in that the number of active genes selected due to noise was excusable, and at most one order of magnitude. Besides, we also chose $q = 0.01$ to obtain a more comprehensive analysis result. We test the performance of enrichment analysis methods for using $q = 0.001, 0.005, 0.01, 0.05, 0.1, 0.2$ in the solving procedures.

Parameter p_1 is closely related with the coverage of active terms, i.e. the proportion of active genes annotated by the active terms. We have $p_1 \geq 0.5$ in enrichment analysis by experience. Here, we selected $p_1 = 0.8$ and $p_1 = 0.5$ as preset values of generating parameter, and performed sensitivity analysis for using $p_1 = 0.1, 0.3, 0.5, 0.7, 0.9$ in the solving precedures.

Parameter p_2 is the probability of peripheral gene being activated via network propagation. We selected $p_2 = 0.3$ and $p_2 = 0.1$ as preset values of generating parameter, and performed sensitivity analysis for using $p_2 = 0.1, 0.3, 0.5, 0.7, 0.9$ in the solving procedures.

Parameter α is a positive number to balance the log-likelihood and the penalization on size of active term set. A larger α makes the model prone to select a fewer number of terms. Here, we set default value $\alpha = 3$ as recommended in [1], and performed the parameter sensitivity analysis for using $\alpha = 0.1, 1, 3, 10, 100, 1000$ in the solving procedures.

We used $p_1 = 0.8, p_2 = 0.3, q = 0.001$ as a default generating parameter combination. For each alternative value of the corresponding parameter, we just replaced the related value and kept other parameters unchanged. The whole workflow of parameter sensitivity analysis is as follows (for clarity, we illustrate the sensitivity analysis of generating parameter $p_1 = 0.5$):

1. We restricted the terms in biological process domain, with number of covered gene between 2 and 500, and then randomly selected 500 terms from this refined term set to obtain an annotation set.
2. For the above annotation set, we randomly selected 5 biological process terms 20 times as the target active term set. For each target active term set, we generated an active gene list using $p_1 = 0.5, p_2 = 0.3, q = 0.001$.
3. The above 20 active gene lists were the model input. We used $p_1 = 0.1, 0.3, 0.5, 0.7, 0.9$ as model solving parameter values, and kept other parameters as $p_2 = 0.3, q = 0.001$, $\alpha = 3$.
4. For each value of parameter p_1, the 20 model outputs were combined to obtain a 2×2 contingency table. Besides, the Bonferroni corrected hypergeometric test p-values were used as the significant scores for these output terms.
5. The area under the precision-recall (AUPR) was computed for each value of parameter p_1.
6. The above procedure was repeated 10 times and the averaged AUPR was returned for each value of parameter p_1.

In the sensitivity analysis, we used *pr.curve* function provided in the R package PRROC (Version: 1.1 from https://cran.r-project.org/web/packages/PRROC/index.html) to compute the AUPR.

2.2 Results

Follow the parameter sensitivity analysis procedure introduced in the previous section, the sensitivity analysis results are shown in Figure S1-S4.

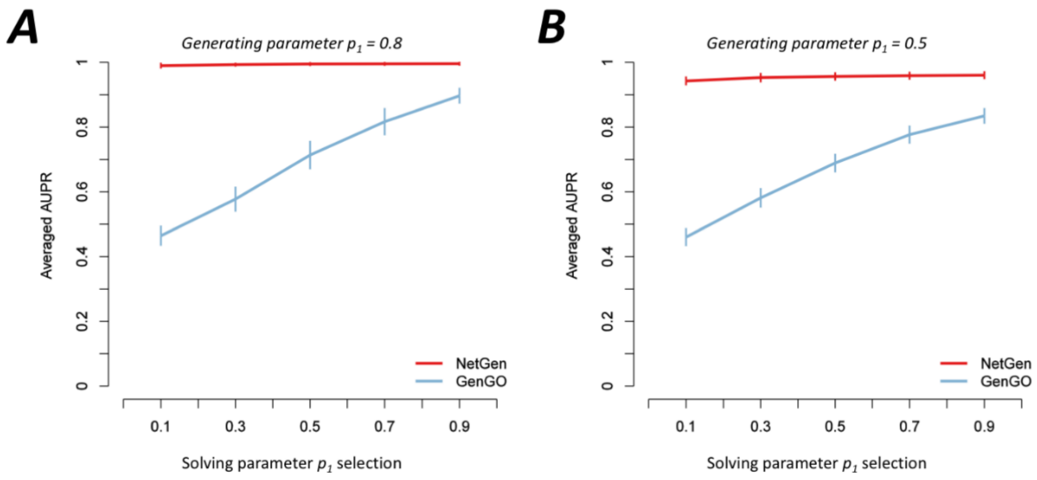

Figure S1: Sensitivity analyses results of p_1. (A) $p_1 = 0.8$ and (B) $p_1 = 0.5$ were selected for generating simulated samples. The performances of NetGen and GenGO were compared using the averaged AUPR plus/minus one-fold standard deviation at different values of solving parameter p_1. The results of NetGen and GenGO were shown in red and blue curve, respectively.

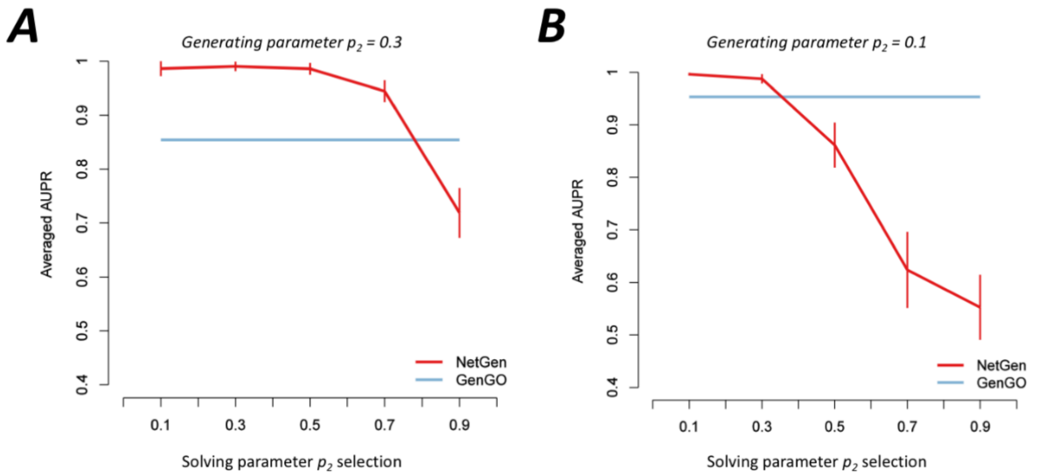

Figure S2: Sensitivity analyses results of p_2. (A) $p_2 = 0.3$ and (B) $p_2 = 0.1$ were selected for generating simulated samples. The performances of NetGen and GenGO were compared using the averaged AUPR plus/minus one-fold standard deviation at different values of solving parameter p_2. The results of NetGen and GenGO were shown in red and blue curve, respectively.

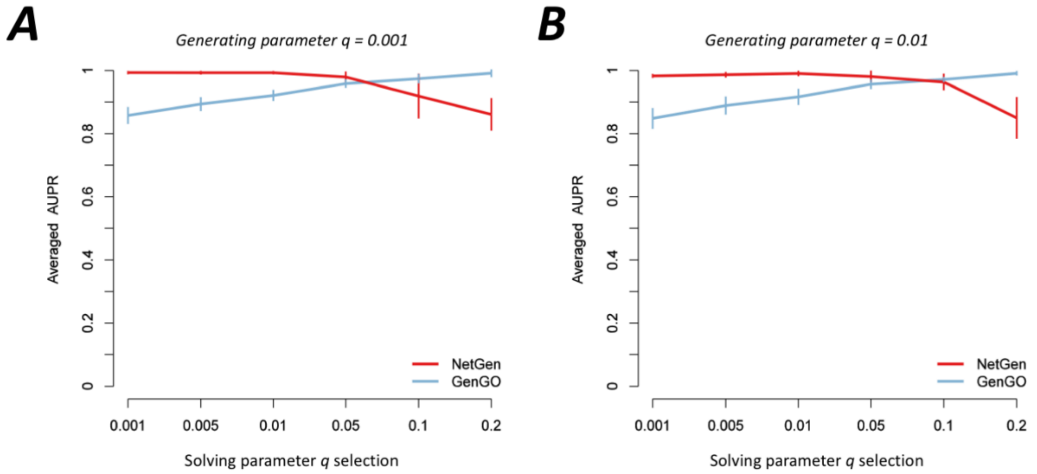

Figure S3: Sensitivity analyses results of q. (A) $q = 0.001$ and (B) $q = 0.01$ were selected for generating simulated samples. The performances of NetGen and GenGO were compared using the averaged AUPR plus/minus one-fold standard deviation at different values of solving parameter q. The results of NetGen and GenGO were shown in red and blue curve, respectively.

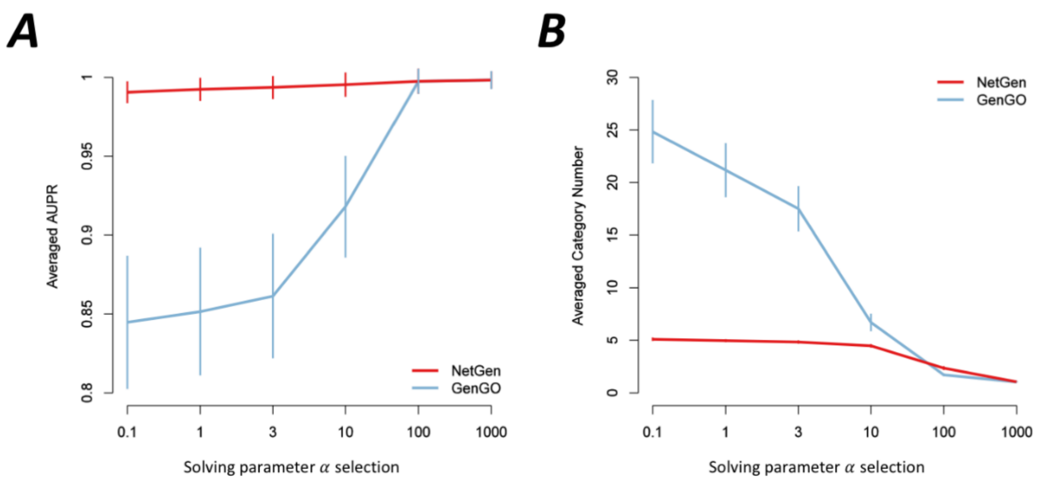

Figure S4: Sensitivity analyses results of α. Both (A) the average AUPR and (B) the average number of identified terms were used to analyze the effect of solving parameter α. The results of NetGen and GenGO were shown in red and blue curve, respectively.

From the results we can see that, NetGen, which consistently maintained a high-level average AUPR and had a lower variance than that of GenGO, was not sensitive to the selection of solving parameter p_1 (Figure S1). However, the average AUPR of GenGO declined when the solving parameter p_1 decreasing. One explicable reason may be that the existence of peripheral genes perturbs the GenGO model, whereas in NetGen model the peripheral genes may assist to identify the true functional terms and counteract the deviation caused by uncertain p_1. Therefore, the network information can help to improve the accuracy of enrichment analysis. The overall results on samples generated by $p_1 = 0.8$ are better than that by $p_1 = 0.5$. This is expected since larger p_1 indicates smaller uncertainty.

Parameter p_2 is the probability of peripheral genes being activated via network propagation, which distinguishes NetGen with other related methods. However, overemphasizing the network information may counter-productive sometimes. Specifically, excessive peripheral genes did confuse the selection of true active terms. The performance of NetGen declined sharply and a larger variance was observed, when the solving parameter was far away from the preset generating value (Figure S2). On the other hand, the maximal distance between solving parameter and the preset true value, to achieve a tolerable performance (average AUPR > 0.95), was roughly about 0.2. This hysteresis can offset the high sensitivity of the inappropriate selection of p_2. The average AUPR maintained a high-level when p_2 was relatively small. Therefore, a relatively conserved strategy was adopted on the selection of p_2 for real data applications. We fixed $p_2 = 0.1$ or $p_2 = 0.05$ in the mixed parameter selection strategy as described in the main text. The result of GenGO was a straight line since GenGO itself is irrelevant with p_2. It is also expected that the result of GenGO on samples generated by $p_2 = 0.3$ is much worse than that by $p_2 = 0.1$.

Parameter q stands for the influence of noise and uncontrollable error in experiment. NetGen performed well when the solving parameter value was around the preset generating value of q, with a lower variance (Figure S3). Similar to the situation in sensitivity analysis of p_2, the curve began to decrease, when the solving parameter value exceeded a tolerable lag. On the contrary, the performance of GenGO did not meet the optimal value of q and had a

continued growth. One reason may be that the active genes generated via network propagation are regarded as noise in GenGO model, which also brings a larger variance.

Parameter α is a positive number to balance the log-likelihood and the size penalization on the active term set. A larger α makes the model prone to select a smaller number of terms. NetGen was not sensitive to the selection of α on both the average AUPR and the average selected term number (Figure S4). Besides, it seems that GenGO prone to identify several redundant terms when $\alpha = 3$. The inferior performance of GenGO may partly be explained by the inappropriate selection of hyperparameter α.

In conclusion, NetGen showed an approximately stable performance to the selection of model parameters. The performance of NetGen was preferable when the model parameters satisfying $p_1 \gg p_2 \gg q$. According to the results of sensitivity analysis, we further designed a mixed parameter selection strategy (see Methods in main text), to fit the practical active gene list in real datasets.

3. Simulation results on other domain

The simulation studies were executed to compare the performance of NetGen with other existing methods. The detailed description of the simulation study can be found in main text. Here, the results on cellular component domain and molecular function domain were shown in Figure S5-S6, and the result on biological process was introduced in Figure 3 in the main text. The results showed that NetGen outperformed other alternative methods on both three domains.

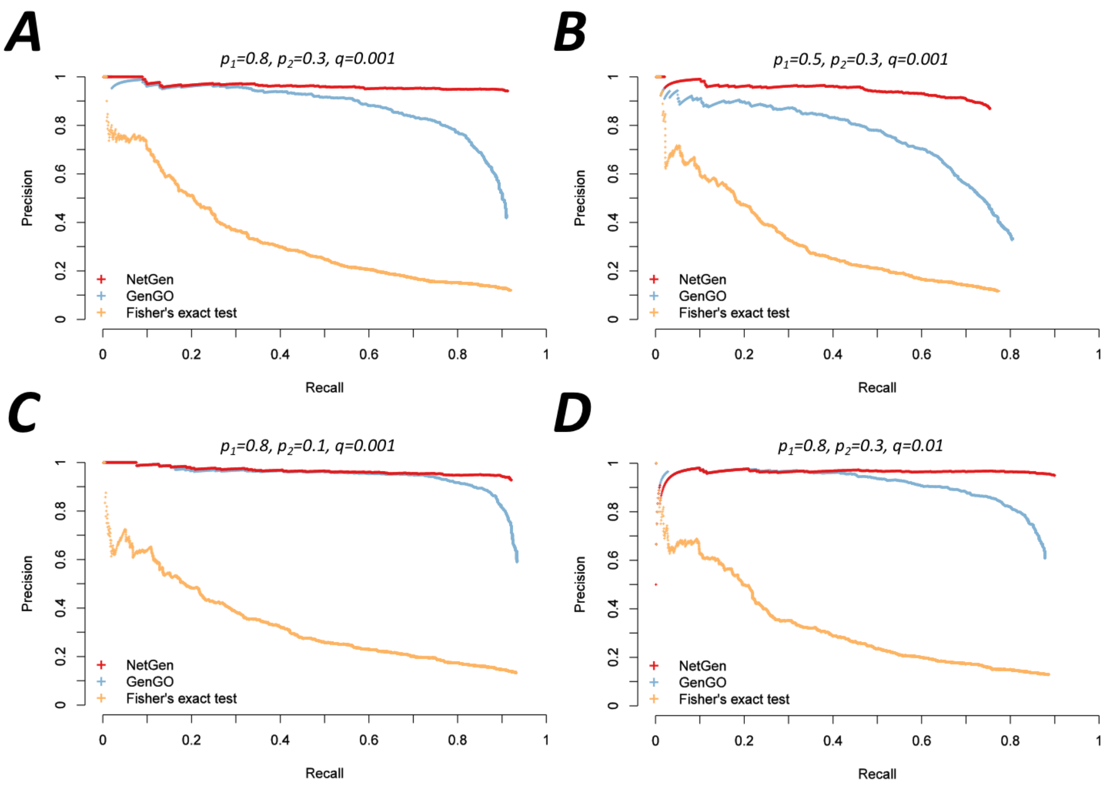

Figure S5. The performance of NetGen and alternative methods on cellular component (CC) domain. Each panel stands for a setting of generating parameters. The performance of NetGen, GenGO and Fisher's exact test are shown in red, blue and orange respectively. The active gene lists were simulated under the assumption of NetGen.

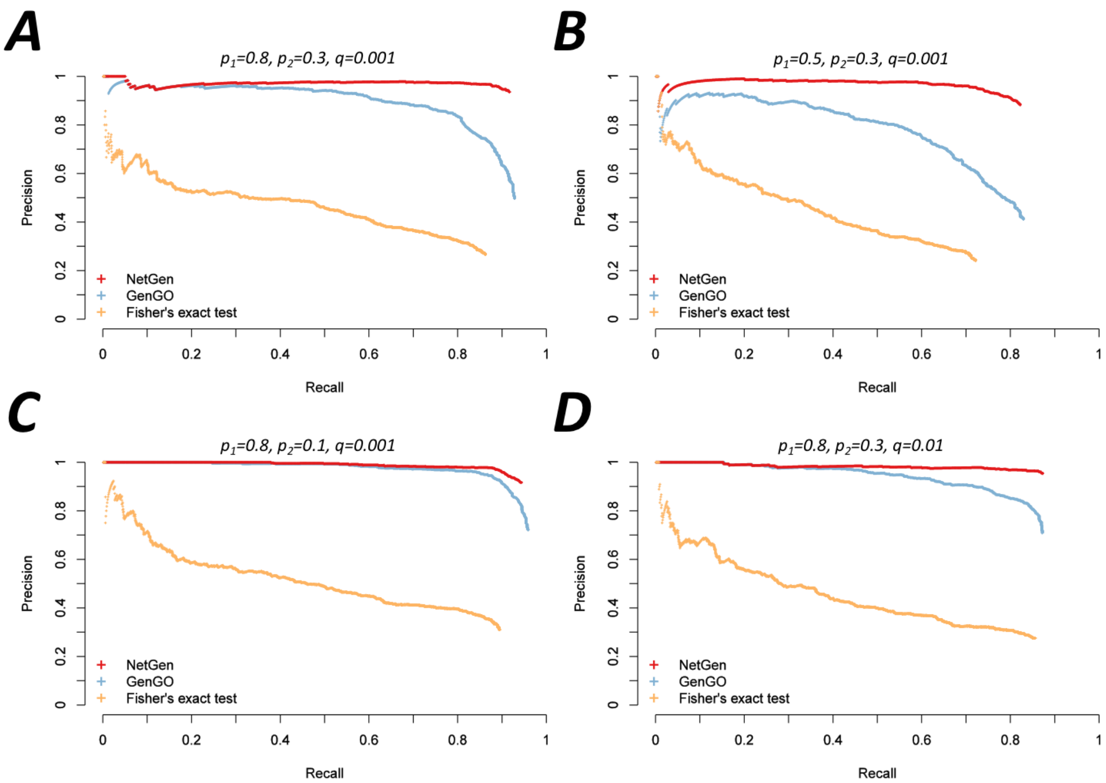

Figure S6. The performance of NetGen and alternative methods on molecular function (MF) domain. Each panel stands for a setting of generating parameters. The performance of NetGen, GenGO and Fisher's exact test are shown in red, blue and orange respectively. The active gene lists were simulated under the assumption of NetGen.

4. Alternative simulation results

In our work, in addition to the simulation procedure as introduced in the main text, we also simulated the circumstance that the active gene lists were unrelated to the network information. Namely, the active gene lists were simulated under the assumption of the GenGO model. The related results can be found in Figure S7-S9.

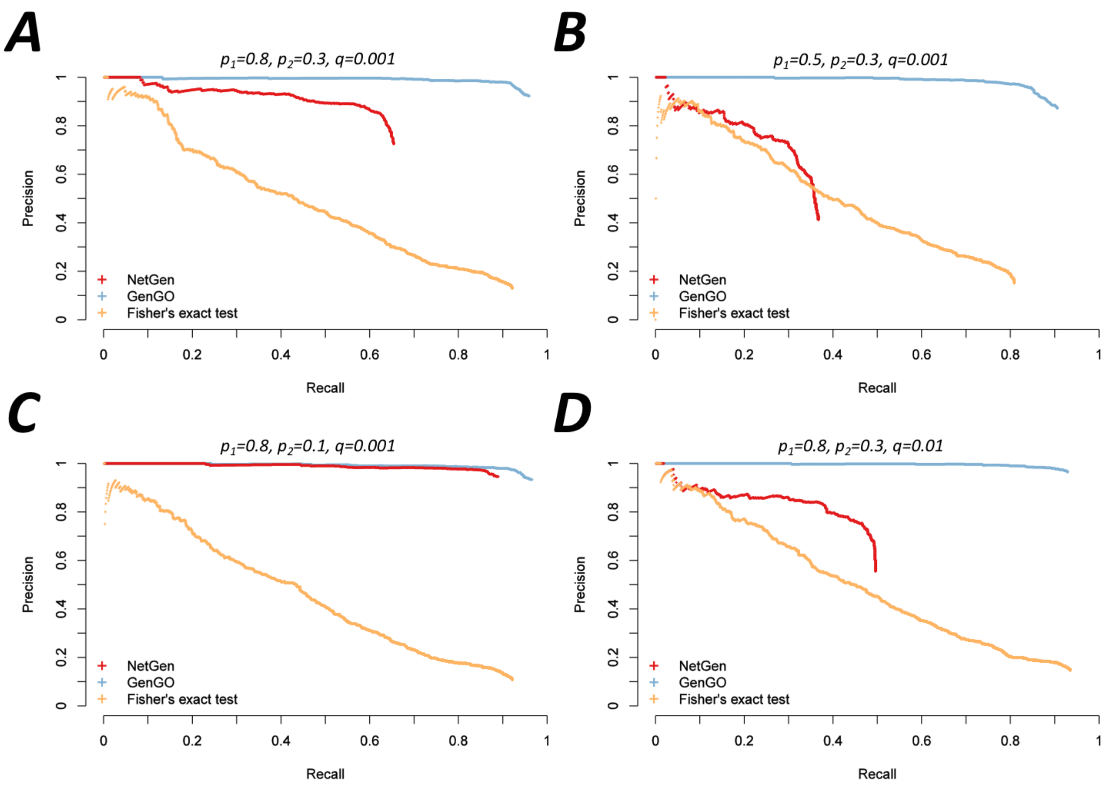

Figure S7. The performance of NetGen and alternative methods on biological process (BP) domain. Each panel stands for a setting of generating parameters. The performance of NetGen, GenGO and Fisher's exact test are shown in red, blue and orange respectively. The active gene lists were simulated under the assumption of GenGO.

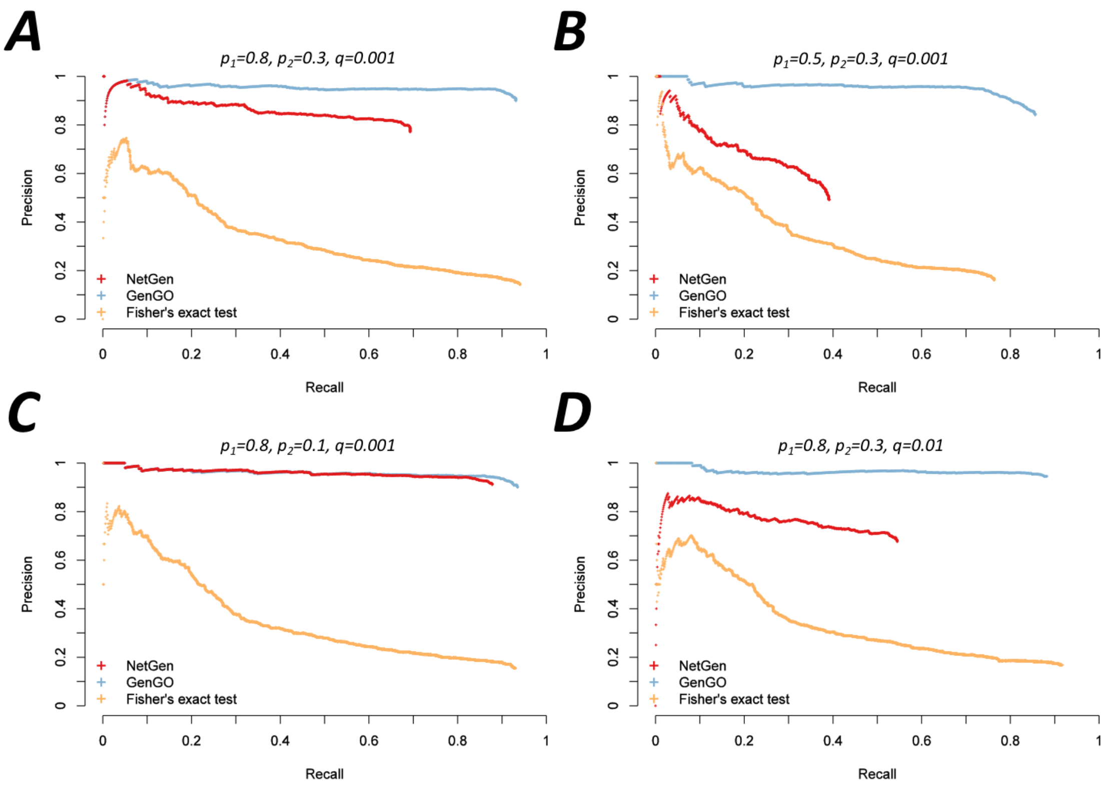

Figure S8. The performance of NetGen and alternative methods on cellular component (CC) domain. Each panel stands for a setting of generating parameters. The performance of NetGen, GenGO and Fisher's exact test are shown in red, blue and orange respectively. The active gene lists were simulated under the assumption of GenGO.

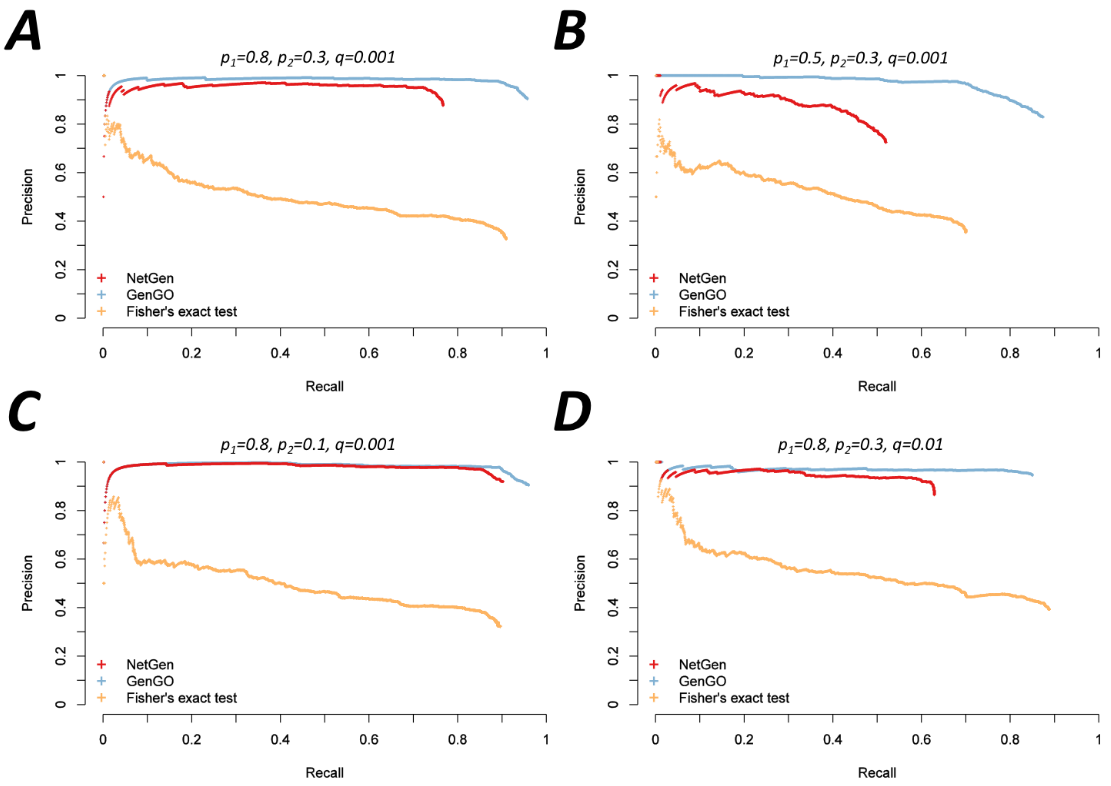

Figure S9. The performance of NetGen and alternative methods on molecular function (MF) domain.

Each panel stands for a setting of generating parameters. The performance of NetGen, GenGO and Fisher's exact test are shown in red, blue and orange respectively. The active gene lists were simulated under the assumption of GenGO.

From the results we can see that the performance of NetGen was satisfactory on both three domains, when the network propagation parameter p_2 is set small enough (Figure S7-S9 C). From a holistic perspective, NetGen achieved a comparable performance with GenGO on the molecular function domain, even if $p_2 = 0.3$ (Figure S9 A and D). This may be directly related with the structure of molecular function domain itself. Not surprisingly, the worst performance occurred when turning down the proportion of the active core genes but keeping a large network propagation parameter p_2. This indeed overly amplified the role of biological network (Figure S7-S9 B), and was absolutely not in conformity with the assumption of GenGO model. On the other hand, overemphasizing both the effect of experiment noisy (q) and network propagation (p_2) made NetGen perform not very well (Figure S7-S8 D). Another notable result was that the lower recall of NetGen, which was related to the small number of identified terms. NetGen cautiously predict candidate terms with a limited number, in order to keep a higher precision. In conclusion, the performance of NetGen is closely related to its particular parameter p_2. According to the sensitivity analysis results (Figure S2), NetGen performed very well even with a p_2 smaller than its true value for generating data. Therefore, we suggested a relatively conserved strategy on the selection of p_2 in practical applications, that is, using a relative small value close to zero, for example 0.05.

5. The description of microarray expression datasets and GO annotation data

5.1 microarray expression datasets

In our work, four microarray gene expression datasets of human complex diseases were selected from the Gene Expression Omnibus (GEO) repository (http://www.ncbi.nlm.nih.gov/geo/, accession number GSE4115, GSE11223, GSE9750, GSE36895, respectively), for real datasets analyses. The selection of microarray expression profiles is based on the following criteria:

1) *Homo sapiens* organism disease.
2) Published (submission date) in recent ten years.
3) Hybridized using Affymetrix Human Genome U133A array or Affymetrix Human Genome U133 Plus 2.0 Array is preferred.
4) A balanced number of case and control samples and the total number is at least 50.
5) Described in or be used by at least one high quality journal paper.

For lung cancer dataset (GSE4115), we combined the original primary and prospective datasets, which made a total of 97 and 90 smokers with and without lung cancer, respectively. For ulcerative colitis dataset (GSE11223), we only used the uninflamed samples in each cohort, which made a 66 ulcerative colitis patients and 69 healthy control donors. As for renal cell carcinoma (GSE36895), the paired expression profiles of 23 clear-cell RCC patients and their related normal cortex were used for further analysis. For all expression datasets, we averaged the expression values of the probes mapping on the same gene. The summaries of the detailed processed datasets are shown in Table S3.

Table S3: The summary of microarray gene expression datasets used in our work.

Dataset	Accession number	#disease	#normal	#genes
Lung cancer	GSE4115	97	90	12493
Ulcerative colitis	GSE11223	66	69	10506
Cervical carcinogenesis	GSE9750	33	24	12494
Renal cell carcinoma	GSE36895	23	23	20108

5.2 GO annotation data

The GO annotation was extracted from R package *org.Hs.eg.db* in Bioconductor project. The detailed information about the GO annotation data was summarized in Table S4.

Table S4. The detailed information about GO annotation data used in this study.

Domain	Number of Terms	Number of annotated genes
Biological process	13226	14614
Cellular component	1510	15505
Molecular function	4090	14237

6. Active gene lists used in the real data applications

To obtain the active gene list of each microarray expression dataset, student's t-test was performed for each gene on the disease and the control cases. We sorted the microarray genes by ascending order of the t-test p-values. The top 100 genes were selected as the differential expression genes, which was then overlapped with the annotated genes. The annotated differential expression genes were used as the final active gene list to perform the functional enrichment analysis.

For each dataset, the annotated differential expression gene set was listed as follows:

Lung cancer (81):

SLC5A1 PRUNE ATP8B1 NSUN3 HDGFRP3 STK38 AGPS TRIM36 DCLRE1C BTD RPL35A SOX9 DND1 C6 TSR1 NNT ZNF160 TFE3 HTRA1 ADH6 PDE8B ZNF611 U2AF2 ECD TMEM110 GOSR2 GTF2H3 SUGP2 MOCS2 PPP2R2D RPL18 P2RX4 NEDD9 SLC4A4 ADK PGF CRY1 EXT2 NOTCH2NL EIF2B3 CORO2A FGF14 DMD DLAT DIP2A USP46 HAUS2 ALPK1 MAN1A2 PPM1D CEP57 DAPP1 PRDX2 NPFFR1 STX3 LAT FBXO9 WWC3 TGDS ARID5A UBQLN4 GNPDA1 RHOQ TNFRSF1A CPE ODF2 PYGB FUT8 ZFR NUDT4 TXN DNAJC6 MTPAP RRAGB ABHD17B IL13RA1 MSH6 MYO1C UNC93B1 MFSD11 KDELR3

Ulcerative colitis (56):

PLCB3 ELL MAPKAPK2 DOCK7 DOHH STK25 TBXA2R INPPL1 C6orf120 APOC1 CEP290 STK35 LARP1 GTF2H5 PPP1R14B SBF1 DIRC2 BRD4 AXIN1 INSR SKIV2L PRCP B3GALT5 TAF12 VPS52 RPS29 ZNF304 C14orf2 ITGA3 GAS6 ARF6 SPSB1 USP54 SLC2A8 GCA CCL11 SERPINF1 FBXL12 TBC1D2B MAN2A1 HIST1H2BN GNB2 ACYP2 ARAF BLVRA HOMER3 PUS1 ACSM1 ADAL C3orf33 GBE1 COMP OXSR1 MVD MLXIP DDX6

Cervical carcinogenesis (94):

PITPNA ZDHHC3 GJA1 SYNGR1 KCTD15 ESR1 AHNAK TRPS1 CDKN2A KANK1 KRT13 KIF18B SYPL1 NAGK MCM6 LMBRD1 UBE2E1 CHMP2B SPRR3 USO1 GINS2 RPL10A NEK2 MCM2 ZNF586 DNMT1 POLD1 RAD54L GOLGA4 CRYL1 GINS1 RPS12 SKP1 SLC24A3 UBE2C MAP2K4 CHAF1B PLCD1 KNTC1 PRDM2 MCM5 ZNF415 TK1 KIF4A KIF2C AURKA CAPN7 TP53AIP1 CCNF LPAR6 SNX3 RPS6KA1 ATP6V1F LAPTM4A PPP2R5A ITM2B DUSP1 NUP62 ATP13A2 RPL29 ATP10D CENPF USP46 LIG1 ARHGAP10 STX7 BBOX1 KLF4 CLCA4 SPAG5 TMEM9B DSC2 RYR1 LANCL1 SYNGR3 AVPR1B TPX2 PSMC3IP SASH1 MAPK10 CDC20 CDT1 CDC45 GIGYF2 TRIM13 TIMELESS GALR3 SLC15A3 IL17RC CDC6 CLCN3 RALB DTL PERP

Renal cell carcinoma (85):

NPHS2 SPAG4 UMOD SFRP1 FGF1 SLC12A1 EGLN3 IGFBP3 ATP6V0D2 HK2 CALB1 GGT6 CWH43 CLDN8 HILPDA HEPACAM2 LPPR1 ATP6V0A4 ACSF2 ANGPTL4 SCNN1G PTH1R CLIC5 FAM3B CLCNKB ENO2 SLIT2 PPAPDC1A PRKCDBP FUT11 CRHBP TMPRSS2 PLCXD3 SAP30 SLC47A2 PTGDS HS6ST2 FXYD4 ATP6V1G3 TYRP1 TCEAL2 TNNC1 DMRT2 CNTN1 HPD SER INA5 KNG1 GPD1L STAP1 C5 CAV1 PDK1 PTPRO RASL11B SLC26A7 GAS1 CAV2 TFAP2B LDHA NPHS1 TCF21 DDB2 SLC2A12 PACRG KCNJ10 DIO1 DACH1 ARHGEF26 GPC3 BMPR1B SEC61G NRK ALDOA VEGFA MUC15 EIF4H CA10 MAN1C1 COL4A6 SOSTDC1 SOST ATP6V1C2 ATP6V1B1 ANGPTL1 FABP5

7. Mixed parameter selection strategy on simulation studies

In real applications, the mixed parameter selection strategy was designed to fit the generating parameters that derive the related active gene list. In this section, we want to test the effectiveness of the mixed parameter selection strategy on the simulation studies.

The workflow of this test is similar to the procedures of simulation studies as introduced in the main text. To execute the mixed parameter selection strategy, each type of the solving parameter combinations was used in the step3 of the simulation study procedure. According to the parameter sensitivity analysis result, we used the alternative value of parameter $p_2 = 0.1$ and $p_2 = 0.05$. The following measurements were used to evaluate the performance of different solving parameter combination.

1) The frequency of the term combinations with the lowest Fisher's exact test p-value;
2) The overall AUPR of the term combinations.

The final results were shown in Figure S10-S11.

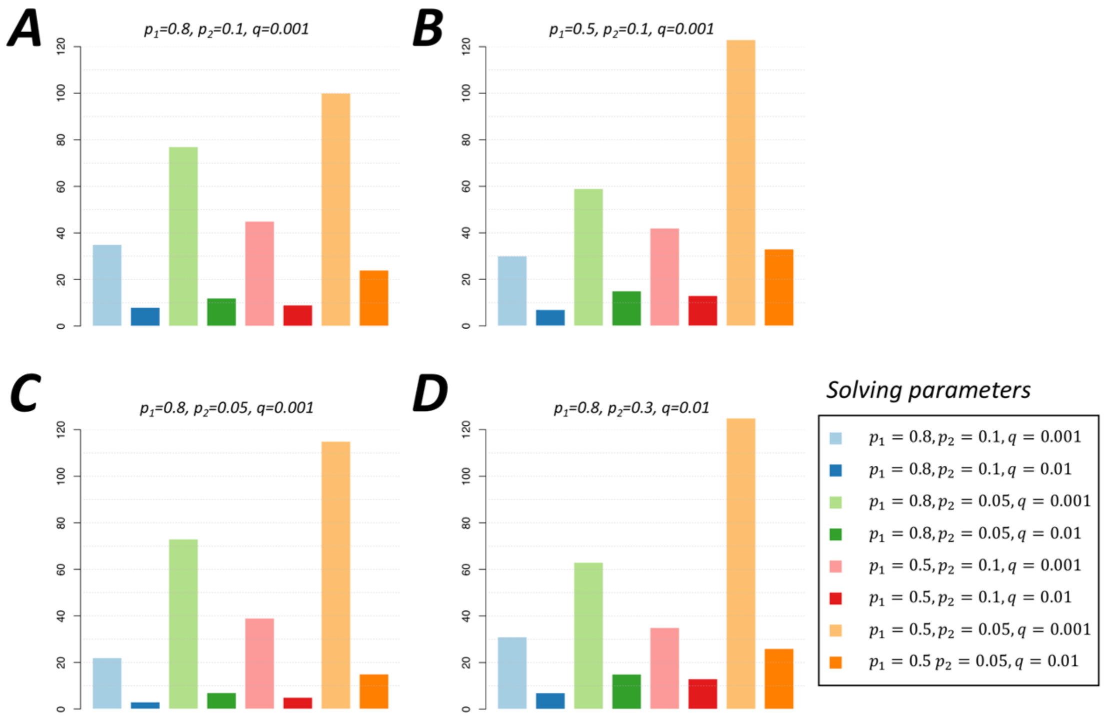

Figure S10. The frequency of the term combinations with the lowest p-value. Each panel stands for a setting of generating parameters. The legend of the related solving parameters is shown in right.

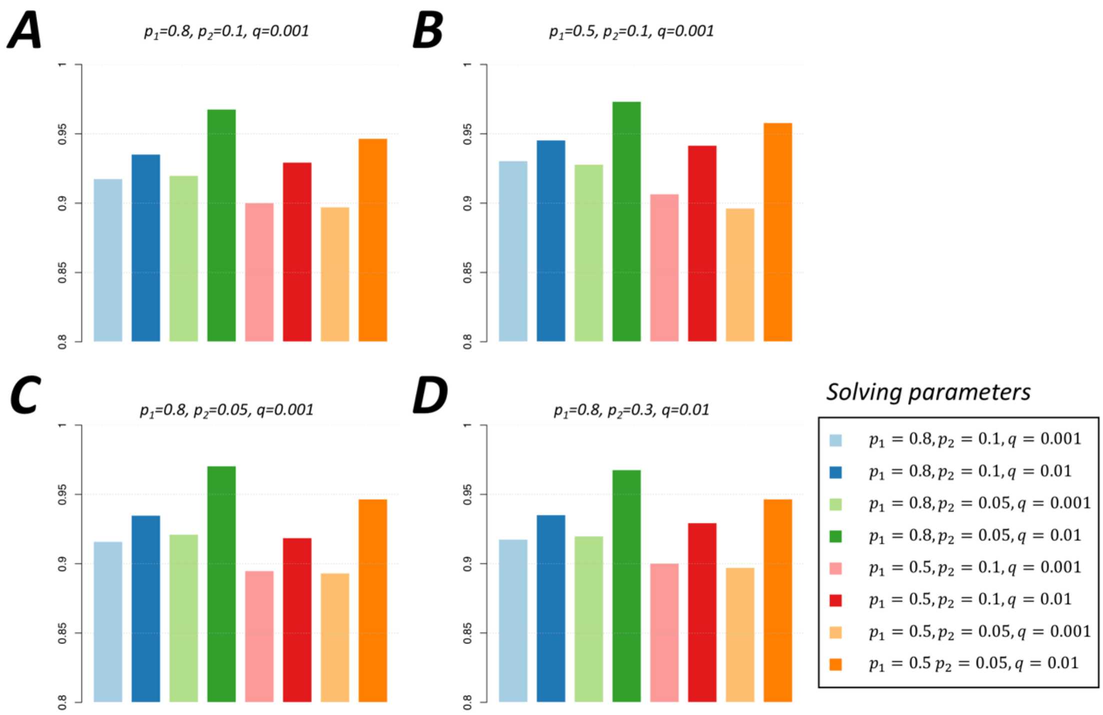

Figure S11. The overall AUPR of the term combinations. Each panel stands for a setting of generating parameters. The legend of the related solving parameters is shown in right.

First, the results in Figure S11 indicate that the generating parameters are not necessarily the best solving parameters. We can observe that the trends are similar in each panel for different generating parameters. The parameter setting $p_1 = 0.8, p_2 = 0.05, q = 0.01$ achieves the best AUPR in all cases. On the other hand, the performances of different solving parameters are very close, which also confirms that the parameters of NetGen are quite robust.

Second, the patterns of Figure S10 and S11 are very different. Under the same parameter combination of p_1 and p_2, a relatively larger q is prone to decrease the Fisher's exact test p value of the found term set. However, a relatively larger q often has better performance in terms of the overall AUPR. This phenomenon implies the Fisher's exact test p-value maybe not a good indicator of the term combination's quality.

Selecting an appropriate solving parameter in real application is very difficult. There may not exist the optimal solving parameter combination. Therefore, we can use the mixed parameter selection strategy to produce multiple solutions in real applications, which offers more information of the underlying biological processes for the downstream analysis.

References

1. Lu Y, Rosenfeld R, Simon I, Nau GJ, Bar-Joseph Z: **A probabilistic generative model for GO enrichment analysis**. *Nucleic Acids Res* 2008, **36**(17):e109.
